# Rift Valley Fever Virus NSs Protein Promotes Post-Transcriptional Downregulation of Protein Kinase PKR and Inhibits eIF2α Phosphorylation

**DOI:** 10.1371/journal.ppat.1000287

**Published:** 2009-02-06

**Authors:** Tetsuro Ikegami, Krishna Narayanan, Sungyong Won, Wataru Kamitani, C. J. Peters, Shinji Makino

**Affiliations:** 1 Department of Microbiology and Immunology, The University of Texas Medical Branch at Galveston, Galveston, Texas, United States of America; 2 Department of Pathology, The University of Texas Medical Branch at Galveston, Galveston, Texas, United States of America; 3 Sealy Center for Vaccine Development, The University of Texas Medical Branch at Galveston, Galveston, Texas, United States of America; 4 Center for Biodefense and Emerging Infectious Diseases, The University of Texas Medical Branch at Galveston, Galveston, Texas, United States of America; University of Washington, United States of America

## Abstract

Rift Valley fever virus (RVFV) (genus *Phlebovirus*, family *Bunyaviridae*) is a negative-stranded RNA virus with a tripartite genome. RVFV is transmitted by mosquitoes and causes fever and severe hemorrhagic illness among humans, and fever and high rates of abortions in livestock. A nonstructural RVFV NSs protein inhibits the transcription of host mRNAs, including interferon-β mRNA, and is a major virulence factor. The present study explored a novel function of the RVFV NSs protein by testing the replication of RVFV lacking the NSs gene in the presence of actinomycin D (ActD) or α-amanitin, both of which served as a surrogate of the host mRNA synthesis suppression function of the NSs. In the presence of the host-transcriptional inhibitors, the replication of RVFV lacking the NSs protein, but not that carrying NSs, induced double-stranded RNA-dependent protein kinase (PKR)–mediated eukaryotic initiation factor (eIF)2α phosphorylation, leading to the suppression of host and viral protein translation. RVFV NSs promoted post-transcriptional downregulation of PKR early in the course of the infection and suppressed the phosphorylated eIF2α accumulation. These data suggested that a combination of RVFV replication and NSs-induced host transcriptional suppression induces PKR-mediated eIF2α phosphorylation, while the NSs facilitates efficient viral translation by downregulating PKR and inhibiting PKR-mediated eIF2α phosphorylation. Thus, the two distinct functions of the NSs, i.e., the suppression of host transcription, including that of type I interferon mRNAs, and the downregulation of PKR, work together to prevent host innate antiviral functions, allowing efficient replication and survival of RVFV in infected mammalian hosts.

## Author Summary

The mosquito-borne bunyavirus Rift Valley fever virus (RVFV) devastates both humans and domestic animals; it causes abortions in ruminants and complications such as hemorrhage, encephalitis, or retinal vasculitis in humans. A major RVFV virulence factor, NSs, disables host cell mRNA synthesis. Here we describe our new evidence that showed NSs working in a second way; in addition to inhibiting host cell transcription, NSs kept translation active in infected cells. It is well-established that activated protein kinase PKR phosphorylates a translation factor, eIF2α, and then this phosphorylated eIF2α suppresses translation. We found that NSs decreased PKR abundance and prevented eIF2α phosphorylation in infected cells, allowing efficient viral translation and replication. In contrast, when cells were infected with an RVFV mutant lacking NSs in the presence of transcriptional inhibitors that mimic the transcription inhibition function of NSs, the PKR reduction did not occur and phoshorylated eIF2α was accumulated, resulting in the inhibition of virus gene expression and replication. Thus, NSs functions in two ways to help RVFV replicate in mammalian hosts: its newly identified PKR downregulation function secures efficient viral translation, and its host transcription inhibition function suppresses the expression of host innate antiviral functions.

## Introduction

Rift Valley fever virus (RVFV) is a mosquito-borne zoonotic pathogen, which is distributed in sub-Saharan Africa [1] and has also caused large outbreaks in Madagascar [2], Egypt [3],[4],[5], Saudi Arabia [6], and Yemen [6]. In endemic areas, RVFV naturally circulates among mosquitoes and ruminants, such as sheep, goat and cattle [7]. RVFV infection in adult ruminants causes febrile illness and a high rate of abortions, while some newborn animals less than 1–2 weeks of age develop an acute infection which results in higher mortality rates than those in adults [8]. Humans infected with RVFV usually develop an acute febrile myalgic syndrome; however, a small percentage of patients have a lethal illness that results in hepatic damage, hemorrhagic fever-like illness, encephalitis and/or retinal vasculitis [8].

Due to the exotic origin of the virus, the potential for the aerosol transmission [9],[10],[11],[12] and serious consequences for humans and livestocks, wild-type (wt) RVFV is classified as a Risk Group 3 pathogen, that needs to be handled in a high-containment facility, e.g., a biosafety level (BSL)-4 laboratory, whereas a highly attenuated MP-12 strain of RVFV, produced after 12 serial passages of wt RVFV ZH548 in MRC-5 cells in the presence of 5-fluorouracil [13], is a Risk Group 2 pathogen.

RVFV, which belongs to the genus *Phlebovirus*, family *Bunyaviridae*, is a negative-stranded RNA virus carrying a single-stranded, tripartite RNA genome composed of S, M and L segments [14]. The S segment encodes N and NSs genes and uses an ambi-sense strategy to express the N and NSs proteins in infected cells; N mRNA encoding N protein is transcribed from the viral-sense (negative-sense) S segment, while NSs mRNA encoding NSs protein is transcribed from the antiviral-sense (positive-sense) S segment. Monocistronic M mRNA and L mRNA are transcribed from the viral-sense M and L segments, respectively. M mRNA has one large open-reading frame (ORF) which encodes the nonstructural NSm protein, a 78-kDa glycoprotein and two major viral structural glycoproteins, Gn and Gc [15],[16]. L mRNA encodes L protein, a viral RNA-dependent RNA polymerase. Both N and L proteins are required for viral RNA synthesis, while Gn and Gc function as envelope proteins [14]. NSm and NSs, both nonstructural proteins, are dispensable for viral replication in cell cultures [17],[18],[19],[20], but are involved in viral pathogenesis [21],[22],[23].

RVFV NSs protein is not essential for virus replication in cell cultures [19],[20], yet plays a critical role in viral virulence [21]. A naturally occurring RVFV mutant Clone 13, which lacks approximately 70% of the NSs gene [20], is highly attenuated in mouse, and, when reassortant viruses between wt RVFV and Clone 13 were characterized, the NSs was revealed as a major determinant of viral virulence in the mouse model [21]. The NSs localizes in the nucleus and cytoplasm in both RVFV-infected cells and NSs-expressing cells; further, the nuclear NSs, but not the cytoplasmic NSs, forms a unique filamentous structure [24]. The NSs suppresses the transcription of host mRNAs by interacting with the p44 subunit of TFIIH, an essential transcriptional factor for cellular RNA polymerase II [25]. Furthermore, the RVFV NSs binds to Sin3A-Associated Protein 30 (SAP30), which is important for maintaining the repressor complex containing histone deacetylase 3 on the interferon (IFN)-β promoter, and suppresses the IFN-β promoter activation early in infection [26]. Accordingly, the NSs protein in the nucleus of infected cells most probably exerts these host transcriptional-suppressive activities, including that of IFN production inhibition, and contributes to viral virulence [21]. In contrast, the biological function of cytoplasmic NSs is largely unknown. RVFV NSs expression promotes RVFV minigenome RNA synthesis driven by N and L protein in expression studies [27]. Because RNA synthesis of bunyaviruses occurs in the cytoplasm, NSs protein in cytoplasm may promote the minigenome RNA synthesis by unknown mechanism.

To establish that RVFV NSs exhibits a novel function, we hypothesized that, when combined, RVFV replication and NSs-induced host transcription suppression likely induces a cellular environment that is unsuitable for viral replication. Thus, to secure efficient RVFV replication, the NSs protein, in turn, alters this putative, virally unfriendly cellular environment to one that supports efficient viral replication. To test this possibility, we examined the replication of RVFV lacking the NSs gene, a mutant which was generated by employing a reverse genetics system [19], in the presence of a host transcription inhibitor, e.g., actinomycin D (ActD) or α-amanitin; these drugs were selected because they mimicked the host transcriptional suppressive activities of the NSs. Consistent with our supposition, RVFV lacking the NSs, but not RVFV, failed to replicate efficiently in ActD-treated cells. We noted that double-stranded RNA (dsRNA)-dependent protein kinase (PKR)-mediated eukaryotic initiation factor (eIF)2α phosphorylation suppressed the translation of RVFV lacking NSs in the presence of ActD. Further studies uncovered that RVFV NSs promoted PKR downregulation as early as 4 hours post-infection, and prevented eIF2α phosphorylation, which secured efficient viral translation. We speculate that this novel function of RVFV is important for counteracting the antiviral activities of PKR and allowing efficient virus replication and survival in infected hosts.

## Results

### RVFV lacking the NSs undergoes poor viral protein synthesis and viral replication in ActD-treated cells

**Figure 1:**
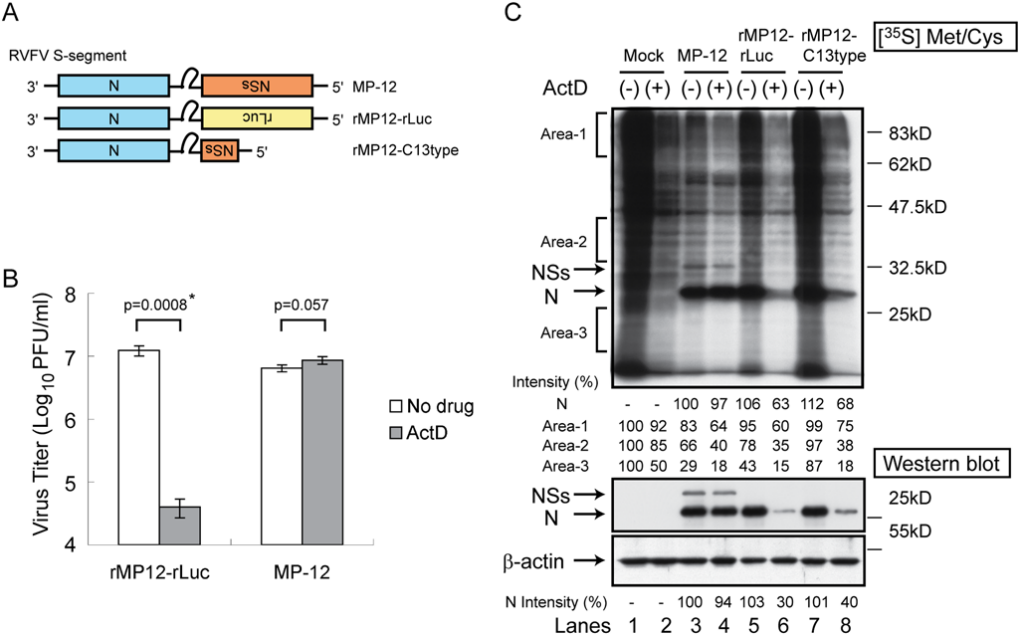
Effects of ActD on the replication and protein synthesis of MP-12 and MP-12 lacking the NSs gene. (A) Schematic representations of the S segments of MP-12, rMP12-rLuc, and rMP12-C13type. (B) Type I IFN-deficient VeroE6 cells were mock-treated or independently infected with MP-12 and rMP12-rLuc at an moi of 3, immediately treated with ActD (5 µg/ml) or untreated, and culture fluids were harvested at 16 h.p.i. Virus titers of MP-12 and rMP12-rLuc were measured by a plaque assay. The virus replication of rMP12-rLuc was significantly reduced in the presence of ActD (*p<0.001; Student's *t*-test). Data are expressed as mean+/−standard deviation of three independent experiments. (C) Vero cells were mock-infected or independently infected with MP-12, rMP12-rLuc, and rMP12-C13type as described above. Cells were radiolabelled with [^35^S] labelled Methionine/Cysteine between 15 h.p.i. and 16 h.p.i. Cell extracts were prepared at 16 h.p.i. and applied to SDS-PAGE (top panel). Intensities of signals in three separate areas (area 1, 2, and 3), which were determined by densitometric analysis, are shown at the bottom of the top panel. Signal intensities of the mock-infected cells were used as the scale to measure the others and were considered as 100%. The intensities of RVFV N proteins were also shown at the bottom of the top panel (N). The signal intensity of N protein in MP-12–infected cells in the absence of ActD was represented as 100%. For Western blot analysis (middle and bottom panels), cell extracts from mock-infected cells and infected cells were prepared at 16 h.p.i., and analyzed in a Western blot in which we used an anti-RVFV antibody (middle panel) and an anti–β-actin antibody (bottom panel). Data are representative of three independent experiments.

To explore a novel biological function of RVFV NSs protein, in addition to its host transcriptional shutoff activity, we investigated the effect of ActD, a host transcriptional inhibitor, on the replication of MP-12 lacking the NSs gene in IFN-incompetent VeroE6 cells. VeroE6 cells were mock-infected or infected with MP-12 or rMP12-rLuc, which expressed *Renilla* luciferase (rLuc) in place of the NSs (Figure 1A) at a multiplicity of infection (moi) of 3. After 1 h virus adsorption, cells were incubated in the absence or presence of 5 µg/ml of ActD. Supernatants were harvested at 16 hours post-infection (h.p.i.), and virus titers were measured by plaque assay. ActD treatment had little effect on MP-12 titers, yet it significantly reduced the titer of rMP12-rLuc (Figure 1B), which suggested that the RVFV NSs was important for an efficient virus replication in the presence of ActD.

To understand how the NSs protein exerted an efficient viral replication in the presence of ActD, we analyzed the status of host and viral translation. VeroE6 cells were mock-infected or independently infected with MP-12, rMP12-rLuc and rMP12-C13type, the latter of which lacks approximately 70% of the NSs ORF (Figure 1A), at an moi of 3. Cells were radiolabelled with [^35^S] Methionine/Cysteine from 15 to 16 h.p.i. Cell extracts were prepared at 16 h.p.i., and the samples were applied to SDS-PAGE (Figure 1C, top panel). In the absence of ActD, the host protein synthesis of rMP12-rLuc-infected cells (Figure 1C, lane 5) and rMP12-C13type-infected cells (Figure 1C, lane 7) was more efficient than that of MP-12-infected cells (Figure 1C, lane 3). It is possible that NSs-mediated transcriptional suppression that occurred only in MP-12-infected cells, but not in cells infected with viruses lacking NSs, caused a reduction of host mRNA abundance, leading to the less efficient translation of host mRNAs in MP-12-infected cells. Efficient N protein synthesis occurred in cells infected with all three viruses in the absence of ActD, a finding which was consistent with our previous studies [19], while for some unknown reasons the accumulation of N proteins of rMP12-rLuc and rMP12-C13type was slightly higher than that of MP-12. ActD treatment resulted in a strong inhibition of both host proteins and N protein synthesis in rMP12-rLuc-infected cells (Figure 1C, lane 6) and rMP12-C13type-infected cells (Figure 1C, lane 8). In contrast, ActD treatment did not inhibit N protein translation in MP-12-infected cells; rather, it moderately inhibited host protein synthesis in MP-12-infected cells (Figure 1C, lane 4). A similar ActD-induced, moderate host protein synthesis inhibition also occurred in mock-infected cells (Figure 1C, lane 2). Western blot analysis of cell extracts at 16 h.p.i. clearly showed that ActD treatment strongly inhibited N protein accumulation in rMP12-rLuc-infected cells and rMP12-C13type-infected cells, but not in MP-12-infected cells (Figure 1C, bottom panels). In summary, ActD treatment had little effect on MP-12 replication, whereas it strongly inhibited the expression of both host and viral proteins in the cells infected with MP-12 lacking the NSs gene, which resulted in poor virus production.

### NSs protein expression promotes viral replication in rMP12-rLuc–infected cells in the presence of ActD

**Figure 2:**
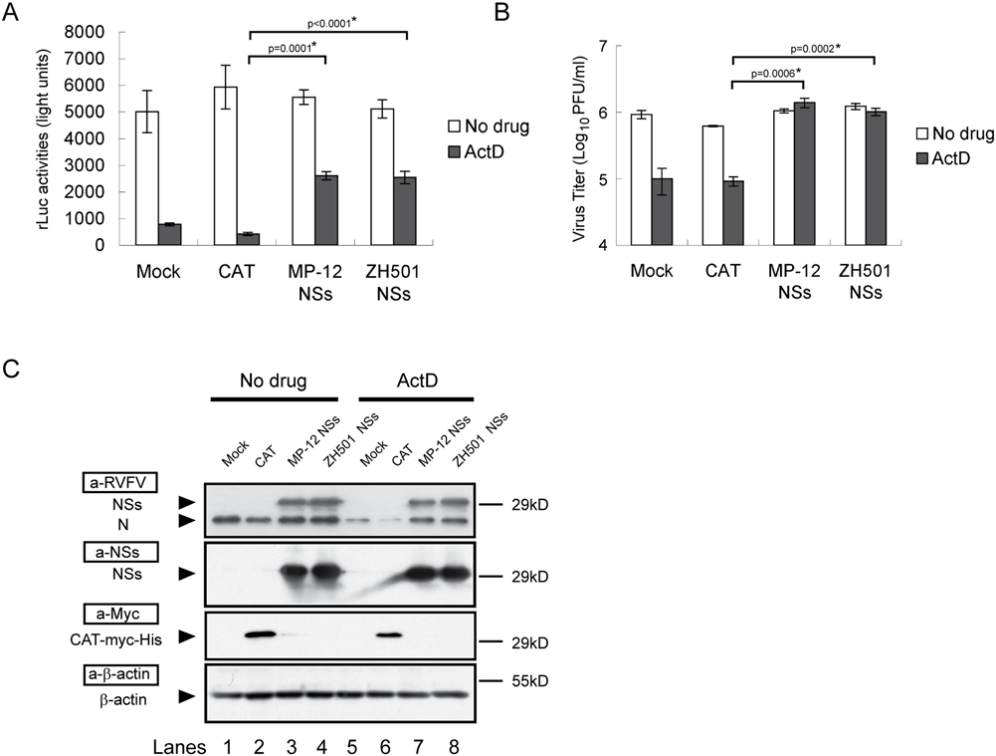
Effects of NSs protein expression on the viral replication of rMP12-rLuc in the presence of ActD. 293 cells were infected with rMP12-rLuc at an moi of 2, transfected with in vitro–synthesized RNA transcripts encoding CAT, MP-12 NSs, or ZH501 NSs, and treated with ActD or were left untreated. Shown are the rLuc activities (A), virus titers (B), and protein accumulations (C) at 16 h.p.i. (A,B) The data were presented as mean+/−standard deviation of three independent experiments, all of which had p values determined by using Student's *t*-test (*p<0.05). (C) The expression levels of N, NSs, CAT-myc-His, and β-actin were determined by Western blotting using anti-RVFV antibody (a-RVFV), anti-NSs antibody (a-NSs), anti-myc antibody (a-Myc), and anti–β-actin antibody (a-β-actin), respectively. The data are representative of three independent experiments.

To further confirm that the NSs exerted an efficient viral replication in the cells that underwent ActD-induced host transcriptional suppression, 293 cells, which showed higher RNA transfection efficiencies than did VeroE6 cells (data not shown), were infected with rMP12-rLuc at an moi of 2. After virus adsorption, infected cells were mock-transfected or independently transfected with in vitro-synthesized RNA transcripts encoding chloramphenicol acetyltransferase (CAT), MP-12 NSs, or wt RVFV ZH501 NSs. Then, the cells were mock-treated or treated with ActD. Analysis of rLuc activities at 16 h.p.i. demonstrated that NSs expression had little effect on rLuc activities in the absence of ActD (Figure 2A). In the presence of ActD, rLuc activities were clearly higher in the cells transfected with MP-12 NSs RNA transcripts or ZH501 NSs RNA transcripts than in those transfected with CAT RNA transcripts or in the mock-transfected samples (Figure 2A). In NSs RNA-transfected cells, similar levels of rMP-12-rLuc titers were observed in both the ActD-treated and mock-treated samples, whereas the rMP12-rLuc virus titers in mock-transfected cells and CAT RNA-transfected cells were significantly lower in the presence of ActD compared to the ActD-untreated cells (Figure 2B). Western blot analysis showed that NSs proteins were indeed expressed in the cells transfected with RNA transcripts encoding MP-12 NSs or wt RVFV ZH501 NSs (Figure 2C, lanes 3, 4, 7, and 8). Also NSs protein expression increased the accumulation of rMP12-rLuc N protein in the presence of ActD (Figure 2C, lanes 7 and 8). These data demonstrated that NSs exerted efficient rMP12-rLuc replication in the presence of ActD. We noted that ActD treatment modestly reduced the accumulation of the CAT protein (Figure 2C, lane 6). Probably large amounts of CAT RNA transcripts that were introduced into the cells partly overcame the translational suppressive effects that were induced by the combination of rMP-12-rLuc replication and ActD treatment.

To test the possibility that ActD treatment alone suppressed translation and RVFV NSs counteracted it, 293 cells were mock-treated or treated with 5 µg/ml of ActD. We examined the resulting polysome profiles at 16 h post–ActD treatment (Figure S1A). Because ActD treatment at 5 µg/ml inhibits the transcription mediated by RNA polymerases I, II and III [28], we expected a reduction in the abundance of cellular mRNAs, tRNAs, and ribosomal RNAs, leading to reduced abundances of polysomes. ActD treatment indeed resulted in a reduced abundance of polysomes, whereas it did not substantially alter the polysome pattern, a finding which suggested to us that ActD treatment did not abolish translational activities. To test the translational competence of the cells treated with transcriptional inhibitors, we transfected 293 cells with in vitro-synthesized RNA transcripts encoding rLuc gene. Cells were mock-treated or treated with ActD or α-amanitin, an RNA polymerase II inhibitor [29]. The rLuc activities at 16 h post-transfection were slightly increased in the cells treated with ActD or α-amanitin (Figure S1B), demonstrating active host translation activities in the presence of either ActD or α-amanitin. These data led to the suggestion that by combining the replication of RVFV lacking the NSs and treatment of ActD, a cellular condition that is unfavorable for translation could be induced and that NSs expression somehow altered the cellular environment from one that was translationally inactive to one translationally active.

### MP-12 replication promotes translation of rLuc mRNA of rMP12-rLuc

**Figure 3:**
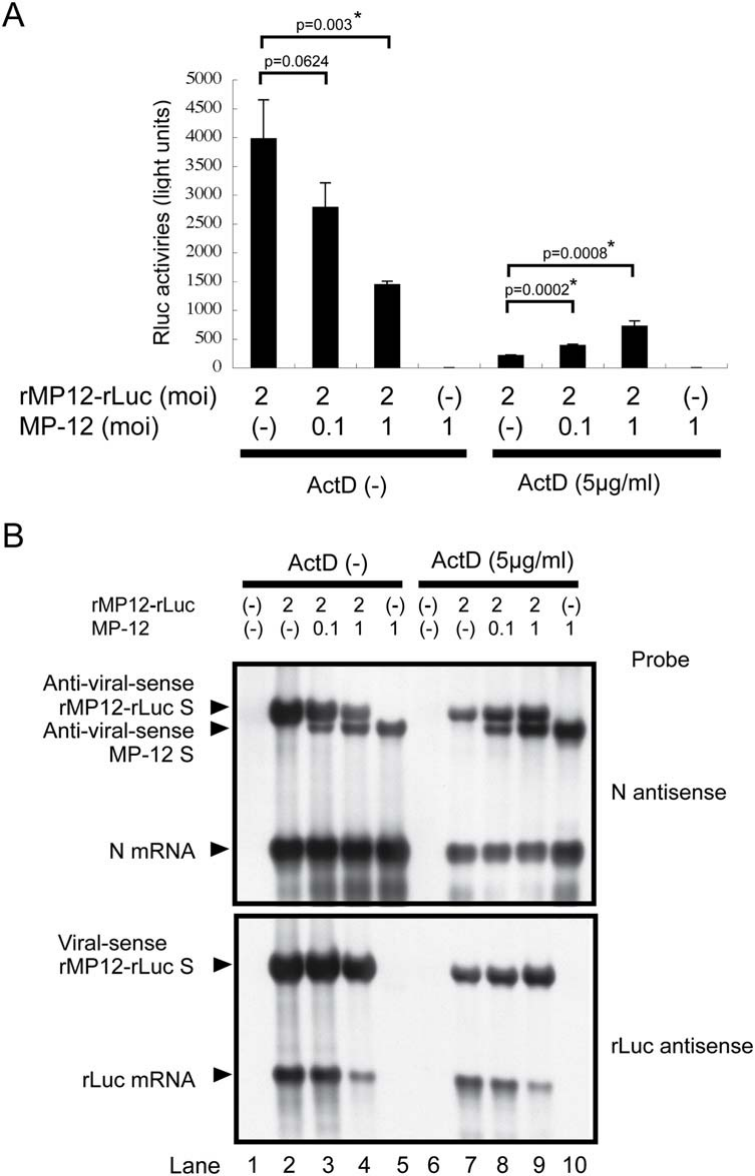
Effect of MP-12 replication on translation of rLuc mRNA of rMP12-rLuc. VeroE6 cells were mock-infected, individually infected with rMP12-rLuc and MP-12, or co-infected with both viruses at the moi indicated in the figure. ActD (5 µg/ml) was added immediately after infection, and cell extracts were harvested at 16 h.p.i. (A) The rLuc activities are presented as mean+/−standard deviation of three independent experiments, for which we determined the p values by using Student's *t*-test (*p<0.05). (B) Northern blot analysis of S-segment RNA, N mRNA, and rLuc mRNA using RNA probe specific to N mRNA and the antiviral-sense S-segment (N antisense) or rLuc mRNA (rLuc antisense). The data are the representative of three independent experiments.

To establish that NSs exerts an efficient viral translation in the presence of a transcription inhibitor, we tested whether coinfection of MP-12 and rMP12-rLuc increases the translation of rLuc mRNA of rMP12-rLuc in the presence of ActD. VeroE6 cells were mock-infected or co-infected with rMP12-rLuc and MP-12; rMP12-rLuc was infected at an moi. of 2, while MP-12 was infected at moi. of 0.1 or 1, as indicated in Figure 3. The cell extracts were harvested at 16 h.p.i., and the rLuc activities (Figure 3A) and accumulation of viral RNAs (Figure 3B) were examined. As expected, ActD-treatment reduced the rLuc activities in cells infected with rMP12-rLuc alone (Figure 3A). Co-infection of MP-12 resulted in the reduction of both rLuc activities (Figure 3A) and the amounts of rLuc mRNA (Figure 3B, lane 4) in the absence of ActD. In the presence of ActD, MP-12 co-infection also reduced the rLuc mRNA abundance (Figure 3B, lane 9), whereas it increased rLuc activities (Figure 3A). The results suggested that NSs protein expressed from MP-12 S-segment promoted an efficient translation of the rLuc mRNA of rMP12-rLuc in the presence of ActD. We also noted that MP-12 co-infection did not reduce the abundance of the viral-sense rMP-12-rLuc S segment in the presence of ActD, and yet it caused the reduction of rLuc mRNA abundance (Figure 3B, lane 9), which we believe implies that NSs expression and ActD treatment generated a cellular environment that was more favorable for rMP-12-rLuc RNA replication than for transcription.

### The status of eIF2α phosphorylation in infected cells

The dsRNAs generated during viral replication activate PKR, which in turn phosphorylates eIF2α [30]. Also 5′-triphosphated single-stranded RNAs activate PKR [31]. The eIF2α is a component of eIF2, which binds to GTP and Met-tRNA to deliver the Met-tRNA to the start codon in capped mRNA, forming a 43S pre-initiation complex [32]. Upon the binding of the 60S ribosomal subunits to the 43S preinitiation complex, eIF2-GDP is released from the ribosome and undergoes a GTP exchange reaction catalyzed by binding with eIF2B, and the resultant eIF2-GTP is recycled for the next round of translation initiation. The phosphorylated eIF2α binds to eIF2B with a high affinity and prevents eIF2B to be used for the subsequent eIF2-GDP-to-eIF2-GTP exchange reaction, leading to the suppression of translation initiation. Hence, the phosphorylation status of eIF2α plays a critical role in translational control [32].

**Figure 4:**
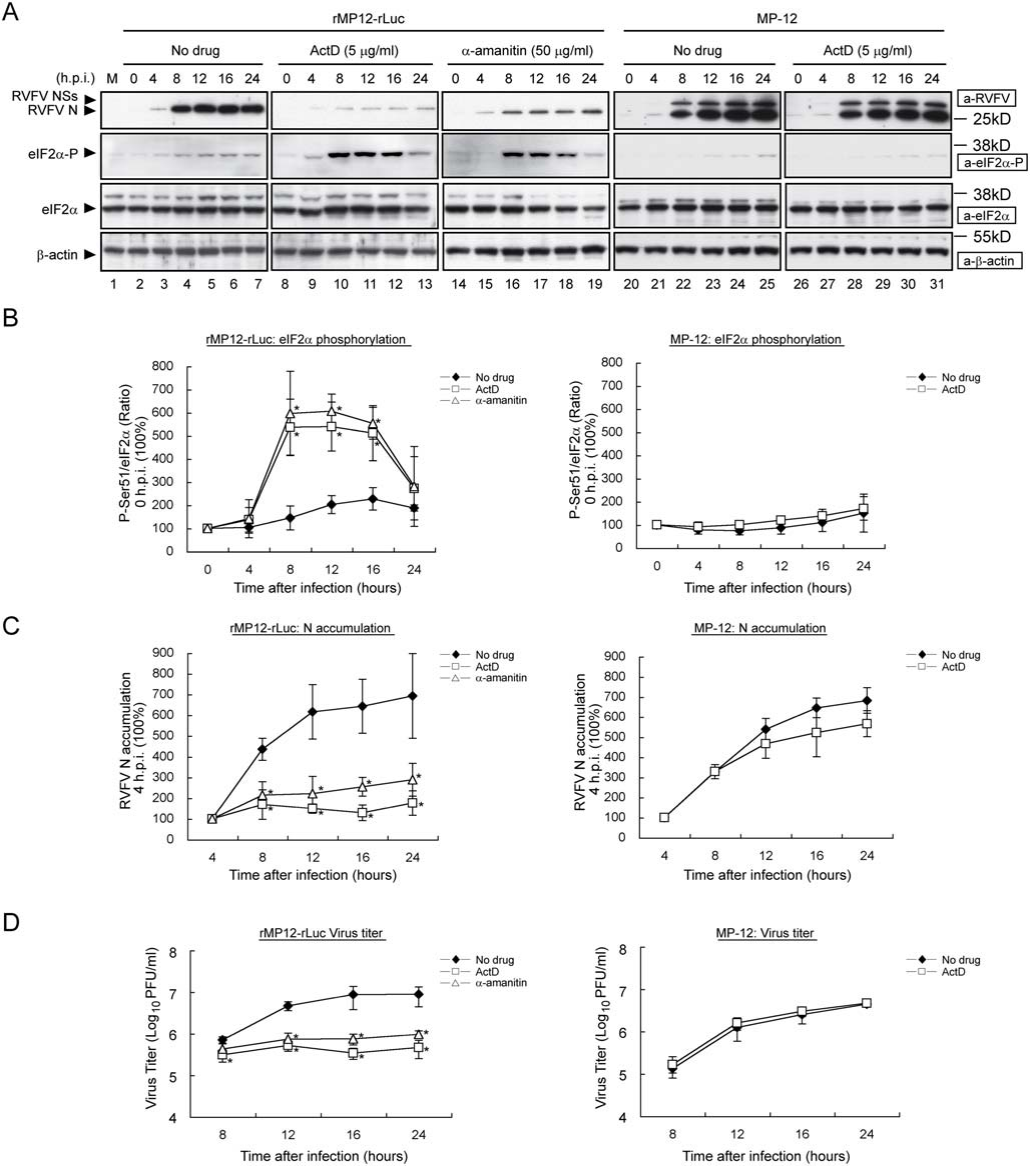
Status of eIF2α phosphorylation in rMP12-rLuc–infected cells and MP-12–infected cells in the presence of transcriptional inhibitors. VeroE6 cells were mock-infected (M) or infected with rMP12-rLuc or MP-12 at an moi of 3, and then immediately treated with ActD (5 µg/ml) or α-amanitin (50 µg/ml), or left untreated (No drug). Samples were harvested at the indicated time points post-infection for Western blot analysis or virus titration. The data shown in the graphs (mean+/−standard deviation) were obtained from three independent experiments with p values determined using Student's *t*-test (*: p<0.05 compared to No drug at each time point). (A) Western blot analyses of RVFV N protein, phosphorylated eIF2α, total eIF2α, and β-actin. The data are representative of three independent experiments. (B) The relative abundance of phosphorylated eIF2α and total eIF2α at various times post-infection. The relative abundance of phosphorylated eIF2α normalized to total eIF2α at 0 h.p.i. is represented as 100%. (C) Accumulation of N protein at various times p.i. The abundance of N protein at 4 h p.i represented as 100% for each of three groups. (D) Kinetics of virus titers in the culture fluids of the infected cells.

We suspected that rMP12-rLuc replication in the presence of ActD may generate dsRNAs and/or 5′-triphosphated single-stranded RNAs, which activate PKR, resulting in the phosphorylation of eIF2α. When we analyzed the level of eIF2α phosphorylation in VeroE6 cells infected with rMP12-rLuc in the absence of transcription inhibitor, we found low levels of eIF2α phosphorylation from 8 to 24 h.p.i. and an efficient accumulation of N protein at 8 h.p.i. and onward (Figure 4A–4C). In contrast, when we treated rMP12-rLuc-infected cells with ActD or α-amanitin, either compound induced an efficient accumulation of phosphorylated eIF2α from 8 to 16 h.p.i., with a concomitant poor N protein accumulation (Figure 4A–4C) and virus replication suppression (Figure 4D). The mechanism of reduction in the abundance of the phosphorylated eIF2α at 24 h.p.i. in the presence of ActD or α-amanitin (Figure 4, lanes 13 and 19) is unknown. When we treated rMP12-rLuc-infected cells with different concentrations of ActD or α-amanitin, we found that the inhibition of phosphorylated eIF2α accumulation was dependent on the concentrations used (Figure S2). Also a strong correlation was seen between an increase in eIF2α phosphorylation and the decrease in both N protein accumulation and infectious virus production (Figure S2). In contrast, no significant accumulation of phosphorylated eIF2α occurred in MP-12-infected VeroE6 cells in the presence of ActD (Figure 4A–4C). As expected, ActD treatment had little effect on MP-12 replication (Figure 4D). These data strongly suggested that the presence of highly phosphorylated eIF2α levels suppressed the translation of viral mRNAs, leading to inefficient virus production, and that the NSs protein somehow suppressed eIF2α phosphorylation, and facilitated efficient viral mRNA translation under the conditions of host transcriptional shutoff.

Because activated caspases 3, 7 and 8 can cleave PKR, releasing the biologically active C-terminus kinase domain from the N-terminus inhibitory domain, resulting in eIF2α phosphorylation [33], we subsequently tested whether the accumulation of phosphorylated eIF2α following the combined activity of viral replication and ActD-treatment was due to induction of apoptosis [34]. VeroE6 cells, either infected with rMP12-rLuc or mock-infected, received the pan-caspase-inhibitor, benzyloxycarbonyl-Val-Ala-DL-Asp(OMe) fluoromethylketone (Z-VADfmk), in the presence of ActD or α-amanitin (Figure S3). Judging from the resulting inhibition of cleaved caspase-3 accumulation in these cells, the Z-VADfmk treatment indeed inhibited apoptosis, whereas it had little effect on the eIF2α phosphorylation status and infectious virus yield. These data suggested to us that the accumulation of phosphorylated eIF2α in cells supporting rMP12-rLuc replication in the presence of ActD or α-amanitin was caspase-independent.

### PKR exerts a critical role in eIF2α phosphorylation in infected cells

**Figure 5:**
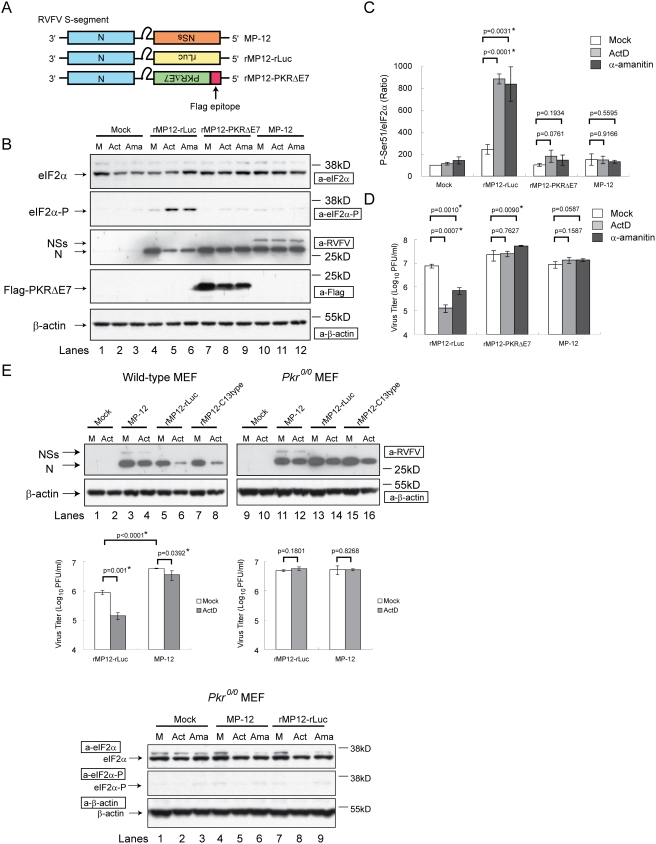
Role of PKR in eIF2α phosphorylation in infected cells under transcriptional suppression. VeroE6 cells (B,C,D), wild-type MEF cells, or *Pkr^0/0^* MEF cells (E) were independently infected with MP-12, rMP12-rLuc, and rMP12-PKRΔE7 at an moi of 3, or were mock-infected. Cells were immediately treated with 5 µg/ml of ActD (Act) or 50 µg/ml of α-amanitin (Ama), or were untreated. Cell extracts were prepared at 16 h.p.i. for Western blot analysis (B,E), and culture fluids were collected for virus titration (D,E). The data shown in the graphs (mean+/−standard deviation) were obtained from three independent experiments with p values of Student's *t*-test (*: p<0.05). The Western blot data is representative of three independent experiments. (A) Schematic representations of S segments of MP-12, rMP12-rLuc, and rMP12-PKRΔE7. (B) Western blot analysis showing the accumulation of eIF2α, phosphorylated eIF2α, N protein, NSs protein, Flag-PKRΔE7, and β-actin in infected VeroE6 cells. (C) Relative abundance of phosphorylated eIF2α and total eIF2α are shown in (B). The relative abundance of phosphorylated eIF2α and total eIF2α in mock-infected, untreated cells represents 100%. (D) Virus titers of rMP12-rLuc, rMP12-PKRΔE7, or MP-12 in VeroE6 cells. (E) Accumulation of RVFV N proteins and β-actin in wild-type MEF cells or in *Pkr^0/0^* MEF cells. M represents mock-infected cells. Middle left panel and middle right panel represent virus titers in wild-type MEF cells and in *Pkr^0/0^* MEF cells, respectively. The bottom panel shows the amounts of total eIF2α, phosphorylated eIF2α and β-actin in mock-infected cells (Mock), MP-12 infected cells, and rMP12-rLuc–infected cells in the presence of 5 µg/ml of ActD (Act), 50 µg/ml of α-amanitin (Ama), or in the absence of the drugs (M).

Four different kinases, including PKR, PKR-like ER kinase (PERK), heme-regulated inhibitor and the general control, non-depressible-2 (GCN2), are known to phosphorylate eIF2α [35]. To determine the role of PKR in the accumulation of phosphorylated eIF2α in rMP12-rLuc-infected cells treated with ActD or α-amanitin, we generated a recombinant MP-12, rMP12-PKRΔE7, which expresses a dominant-negative form of PKR, PKRΔE7 [36] carrying the N-terminal Flag epitope tag, in place of the NSs (Figure 5A). If the replication of MP-12 lacking the NSs gene in cells subjected to host transcriptional suppression activates PKR, which in turn phosphorylates eIF2α, then the virally-encoded PKRΔE7 in rMP12-PKRΔE7-infected cells would interfere with the PKR function, resulting in the inhibition of PKR-mediated eIF2α phosphorylation and thereby leading to efficient viral translation and virus production. VeroE6 cells were mock-infected or independently infected with rMP12-rLuc, rMP12-PKRΔE7 and MP-12 at an moi of 3. After the removal of the inocula, cells were treated with ActD or α-amanitin, and cell extracts were harvested at 16 h.p.i. As expected, rMP12-rLuc replication in the presence of ActD or α-amanitin induced eIF2α phosphorylation, resulted in reduced virus replication (Figure 5B–5D). In contrast, efficient N protein accumulation and efficient virus replication, with no significant accumulation of phosphorylated eIF2α occurred in both MP-12-infected cells and rMP12-PKRΔE7-infected cells in the presence of ActD- or α-amanitin (Figure 5B–5D). This finding strongly suggested that PKR is important for eIF2α phosphorylation in cells infected with the RVFV lacking the NSs in the presence of transcription inhibitors.

To further confirm these data, viral protein accumulation and replication were analyzed in wt mouse embryonic fibroblast (MEF) cells and in *Pkr^0/0^* MEF cells lacking a functional PKR expression [37]. MP-12 efficiently replicated in both wt MEF and *Pkr^0/0^* MEF cells, and ActD treatment had little effect on N protein accumulation and virus replication (Figure 5E, top and middle panels). rMP12-rLuc replication was not as efficient as MP-12 in wt MEF cells in the absence of ActD for an as yet unidentified reason (Figure 5E, middle panel). In ActD-treated wt MEF cells, both rMP-12-C13type and rMP12-rLuc failed to efficiently accumulate N proteins, and rMP-12-rLuc replicated poorly, whereas rMP-12-rLuc underwent efficient N protein accumulation and viral replication in ActD-treated *Pkr^0/0^* MEF cells (Figure 5E, top and middle panels). Furthermore, accumulation of phosphorylated eIF2α did not occur in *Pkr^0/0^* MEF cells that were infected with rMP12-rLuc in the presence of transcriptional inhibitors (Figure 5E, bottom panel). These data were consistent with a notion that PKR triggered the accumulation of phosphorylated eIF2α in cells infected with the MP-12 lacking the NSs, under the conditions of cellular transcriptional suppression, and the NSs protein interfered with the PKR-mediated eIF2α phosphorylation (Figure 5B–5D).

### Testing the possible NSs-mediated suppression of PKR phosphorylation

**Figure 6:**
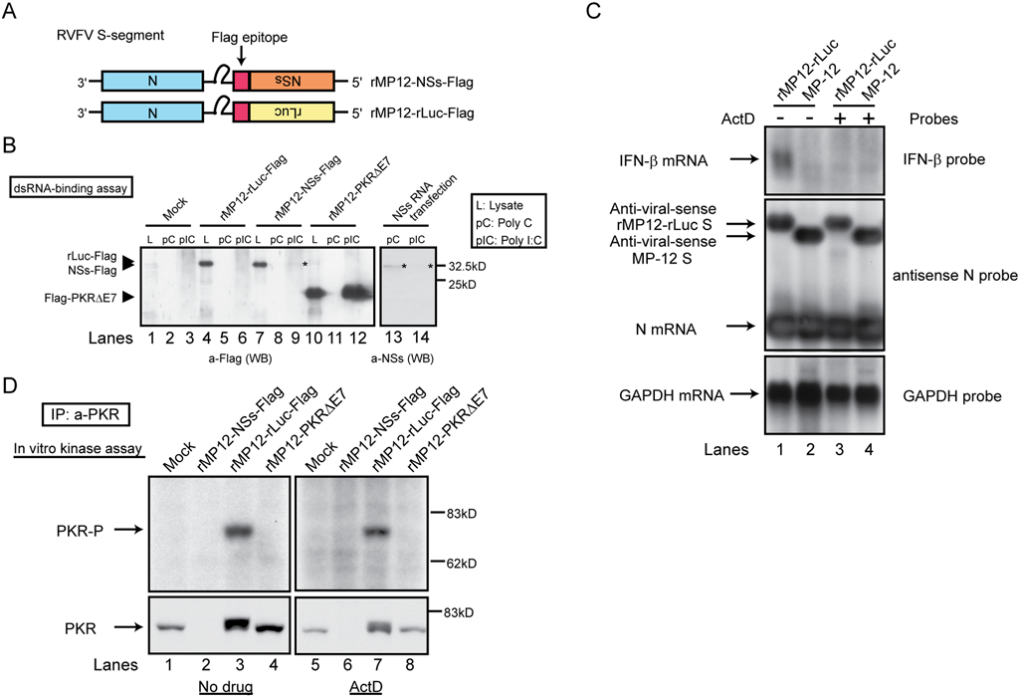
Testing dsRNA binding activity of NSs protein and autophosphorylation of PKR in infected cells. (A) Schematic representations of RVFV S segments of rMP12-NSs-Flag and rMP12-rLuc-Flag. (B) 293 cells were mock-infected (Mock) or infected with rMP12-rLuc-Flag, rMP12-NSs-Flag, or rMP12-PKRΔE7 at an moi of 3 (left panel). In the right panel, 293 cells were transfected with in vitro–synthesized RNA transcripts encoding RVFV MP-12 NSs. At 16 h.p.i. or 16 h post-transfection, cytoplasmic lysates (L) were incubated with poly C (pC) beads or poly I∶C (pIC) beads. The dsRNA binding activity of the NSs was analyzed as described in Materials and Methods. Proteins bound to beads were analyzed by Western blotting using anti-Flag antibody (left panel) or anti-NSs antibody (right panel). Asterisk (*) represents the NSs. (C) 293 cells were infected with rMP12-rLuc or MP-12 at an moi of 3. After infection, cells were mock-treated (−) or immediately treated with ActD (+) at 5 µg/ml. Total RNA was harvested at 8 h.p.i., and accumulations of IFN-β mRNA, antiviral-sense S segment RNA, N mRNA, and GAPDH mRNA were analyzed by Northern blot. (D) 293 cells were mock-infected or infected with rMP12-NSs-Flag, rMP12-rLuc-Flag, or rMP12-PKRΔE7 at an moi of 3, and, then, cells were mock-treated (No drug) or immediately treated with 5 µg/ml of ActD. A cytoplasmic fraction was collected at 16 h.p.i. and the IP-kinase assay of PKR was performed as described in Materials and Methods (top panel). A part of samples was used for Western blot analysis by using anti-PKR monoclonal antibody to show the abundance of immunoprecipitated PKR (bottom panel). Data are representative of two independent experiments (B–D).

To know how the NSs suppressed the eIF2α phosphorylation activity of the PKR function, a dsRNA-binding assay was performed to test the possibility that the NSs binds to dsRNA, sequesters dsRNA from PKR, and interferes with the dsRNA-mediated PKR activation. 293 cells were mock-infected or infected with rMP12-NSs-Flag carrying Flag-tagged NSs, rMP12-rLuc-Flag carrying Flag-tagged rLuc (Figure 6A) or rMP12-PKRΔE7 (Figure 5A). In a separate experiment, 293 cells were transfected with in vitro-synthesized RNA transcripts encoding NSs. Lysates were prepared at 16 h.p.i. or 16 h post-transfection, and incubated with poly I∶C beads (dsRNA) or poly (C) beads (ssRNA). Then the dsRNA-bound complexes were analyzed by a Western blot in which we used an anti-Flag antibody or anti-NSs antibody (Figure 6B). As expected, dsRNA bound to PKRΔE7 [36], whereas it poorly bound to the NSs from rMP12-NSs-Flag-infected cells and that from the NSs-expressing cells (Figure 6B), which suggested that NSs did not suppress PKR activation by its binding to dsRNA.

Because activated PKR undergoes a structural alteration and autophosphorylation [30], we tested whether NSs prevented PKR autophosphorylation. 293 cells were infected with rMP12-NSs-Flag, rMP12-rLuc-Flag or rMP12-PKRΔE7, and mock-treated or immediately treated with ActD. Cell lysates were harvested at 16 h.p.i., and PKR was immunoprecipitated by anti-human PKR antibody, and the PKR bound to the protein A beads was subjected to an immunoprecipitation (IP)-kinase assay by using [γ-^32^P]ATP. We used 293 cells for this assay, because anti-human PKR antibody efficiently immunoprecipitated human PKR in 293 cells, but not non-human primate PKR in VeroE6 cells (data not shown). PKR is induced by IFN-α/β treatment [38] and the abundance of PKR could affect the results of the IP-kinase assay. We suspected that replication of MP-12 mutants lacking NSs may induce IFN-β production, leading to PKR induction [38], whereas ActD treatment prevented the IFN-β production [39]. Indeed, IFN-β mRNA accumulation occurred at 8 h.p.i. in rMP-12-rLuc-infected cells in the absence of ActD, but not in the presence of ActD (Figure 6C), a finding which suggested that ActD treatment inhibited the transcriptional induction of PKR which was induced by the type I IFN in infected 293 cells. We noted an efficient accumulation of rMP12-rLuc N mRNA at 8 h.p.i. in the presence of ActD (Figure 6C). Because rMP-12-rLuc replication in the presence of ActD did not induce an accumulation of phosphorylated eIF2α early in the course of the infection (Figure 4A), this efficient rMP12-rLuc replication probably occurred prior to 8 h.p.i. in the presence of ActD. As shown in Figure 6D, immunoprecipitated PKR from rMP12-rLuc-Flag-infected cells was phosphorylated both in the presence and absence of ActD, which led us to suggest that rMP12-rLuc-Flag replication activated PKR. In contrast, PKR phosphorylation did not occur in mock-infected cells or cells infected with rMP12-NSs-Flag or rMP12-PKRΔE7 (Figure 6D). Most probably, a dominant-negative PKRΔE7 suppressed PKR activation and prevented PKR phosphorylation [36]. To determine why we failed to detect the presence of PKR autophosporylation in rMP12-NSs-Flag-infected cells, we examined the amounts of the immunoprecipitated PKR in these samples (Figure 6D, bottom). Strikingly, substantial reductions in the abundance of cytoplasmic PKR occurred only in the cells supporting rMP12-NSs-Flag replication both in the presence and absence of ActD.

### RVFV NSs promotes PKR downregulation

**Figure 7:**
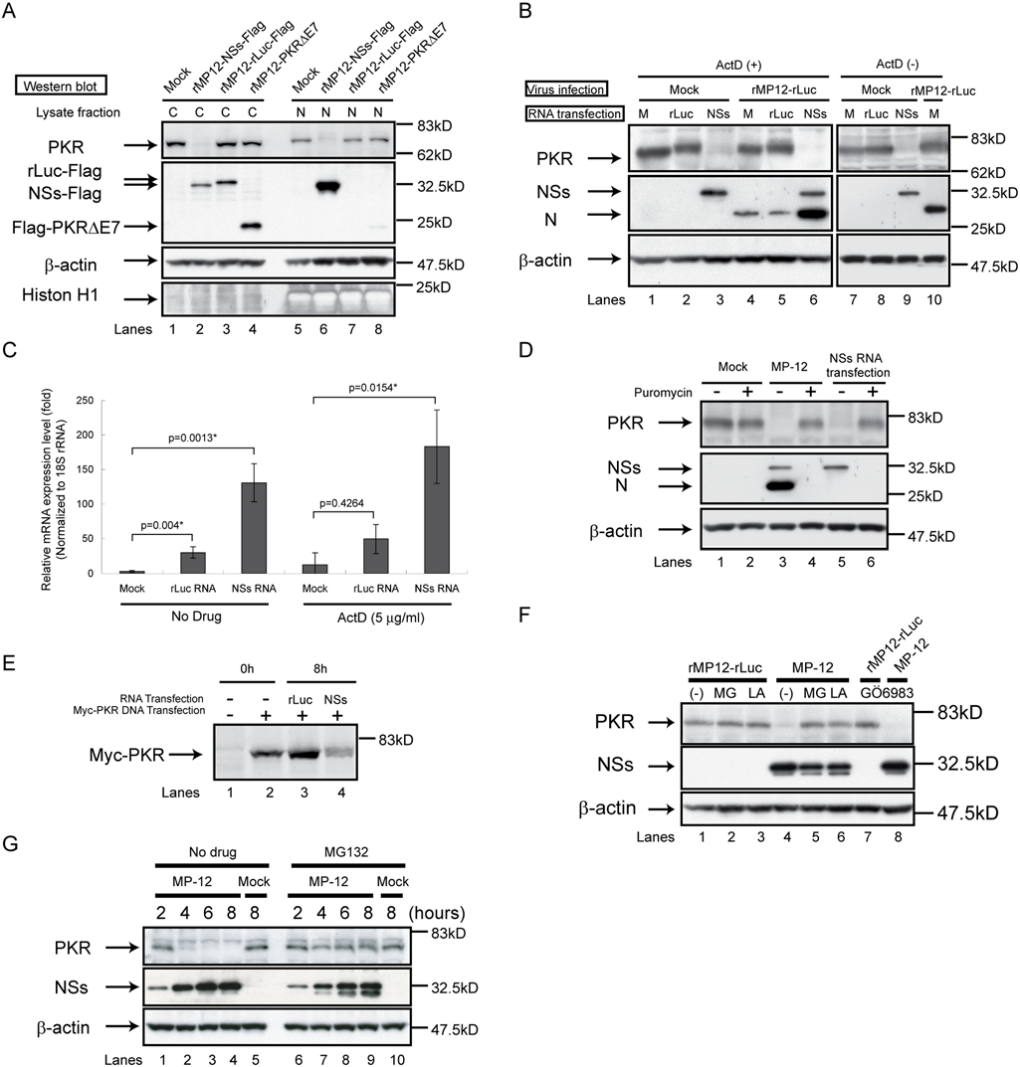
Analysis of NSs-induced PKR downregulation. (A) 293 cells were mock-infected (Mock) or independently infected with rMP12-NSs-Flag, rMP12-rLuc-Flag, and rMP12-PKRΔE7 at an moi of 3. Cells were immediately treated with 5 µg/ml of ActD, and both cytoplasmic (C) and nuclear (N) fractions were collected at 16 h.p.i. Results are shown of Western blot analysis performed by using anti-PKR antibody, anti-Flag antibody, anti–β-actin antibody, and anti-Histone H1 antibody. (B) 293 cells were mock-infected (Mock) or infected with rMP12-rLuc at an moi of 3. Then cells were mock-transfected (M) or transfected with in vitro–synthesized RNA transcripts encoding rLuc (rLuc) or NSs (NSs). Cells were then treated with 5 µg/ml of ActD. Whole-cell lysates were collected at 16 h.p.i. (left panel). Another set of cells were mock-transfected or transfected with in vitro–synthesized RNA transcripts encoding NSs or rLuc, and cell extracts were harvested at 8 h post-transfection (right panel). Western blot analysis was performed by using anti-PKR antibody, anti-RVFV antibody, and anti–β-actin antibody. Lane 10 represents the cell lysate of rMP12-rLuc–infected cells harvested at 8 h.p.i. in the absence of ActD. (C) 293 cells were mock-transfected or transfected with in vitro–synthesized RNA transcripts encoding MP-12 NSs or rLuc. Cells were mock-treated or treated with 5 µg/ml of ActD. Total RNA was harvested at 8 h post-transfection, and analyzed by real-time PCR. The relative abundance of PKR mRNA of each sample was calculated by the ΔΔC_T_ method based on the abundance of 18S ribosomal RNA. The data shown in the graph (mean+/−standard deviation) were obtained from three independent experiments. The p value was determined by Student's *t*-test (*: p<0.05). (D) 293 cells were mock-infected (Mock) or infected with MP-12 at an moi of 3 or transfected with in vitro–synthesized RNA transcripts encoding NSs. Cells were then mock-treated or treated with 100 µg/ml of puromycin. Cell extracts were harvested at 16 h.p.i. or 16 h post-transfection, and the abundance of PKR and viral proteins were analyzed by Western blotting with anti-PKR antibody (top panel), anti-RVFV antibody (middle panel), or anti–β-actin antibody (bottom panel). (E) 293 cells were mock-transfected or transfected with pcDNA3.1-Myc-PKRK296R. Cells were radiolabelled with [^35^S] Methionine/Cysteine between 14 and 16 h post-transfection. Then, cells were harvested (0 h), or transfected with in vitro–synthesized RNA transcripts encoding rLuc or MP-12 NSs. At 8 h post–RNA transfection, cells were harvested (8 h). Radiolabelled myc-tagged PKR was immunoprecipitated by anti-myc monoclonal antibody, analyzed by SDS-PAGE with 7.5% poryacrylamide gel, and visualized by autoradiography. (F) 293 cells were infected with rMP12-rLuc or MP-12 at an moi of 3, and, then, treated with 10 µM of MG132 (MG) or 50 µM of lactacystin (LA) or they were mock-treated (−). Whole-cell lysates were collected at 8 h.p.i., and the abundance of PKR, NSs and β-actin were examined by Western blot analysis. (G) 293 cells were mock-infected (Mock) or infected with MP-12 (MP-12) at an moi of 3, and treated with MG132 (MG132) at 10 µM or they were untreated (no drug used). Whole-cell lysates were collected at 2, 4, 6, and 8 h.p.i. Anti-PKR antibody, anti-NSs antibody, and anti–β-actin antibody were used to detect PKR, NSs, and β-actin, respectively (F and G). Data are representative of two to three independent experiments (A–G).

We subsequently tested the possibility that the NSs downregulated PKR expression or sequestered PKR into the nuclear compartment, leading to the suppression of eIF2α phosphorylation. 293 cells were mock-infected or infected with rMP12-NSs-Flag, rMP12-rLuc-Flag or rMP12-PKRΔE7, treated with ActD, and cytoplasmic and nuclear fractions of cell extracts were prepared at 16 h.p.i. Western blot analyses showed the presence of RVFV NSs both in the cytoplasmic and nuclear fractions, while rLuc and PKRΔE7 signals were observed only in the cytoplasmic fraction. A substantial reduction in PKR abundance occurred in both the cytoplasmic and nuclear fractions of cells infected with rMP-12-NSs-Flag, but not in mock-infected cells and in those infected with rMP12-rLuc-Flag or rMP12-PKRΔE7 (Figure 7A), demonstrating that NSs induced the PKR downregulation in the infected cells. The PKR downregulation also occurred in MP-12-infected VeroE6 cells and in MRC-5 cells that were infected with the wt ZH501 strain of RVFV (data not shown).

To determine whether NSs expression alone induces PKR downregulation, 293 cells were mock-infected or infected with rMP12-rLuc, immediately transfected with in vitro-synthesized RNA transcripts encoding rLuc or NSs, and treated with ActD (Figure 7B, left panel). Cells were harvested at 16 h post-transfection. The clear reduction in the PKR abundance occurred in mock-infected cells expressing NSs, but not in those expressing rLuc (Figure 7B, left panel), demonstrating that NSs protein alone exerted the PKR downregulation.

We subsequently examined the requirement of the ActD treatment for the PKR downregulation. 293 cells were transfected with in vitro-synthesized RNA transcripts encoding NSs or rLuc in the absence of ActD. Analysis of cell extracts harvested at 8 h post-transfection showed the PKR downregulation in the NSs-expressing cells (Figure 7B, right panel), demonstrating that the NSs-mediated PKR downregulation occurred in the absence of ActD.

To further understand the mechanism of the NSs-mediated PKR downregulation, we examined whether NSs expression promoted degradation of PKR mRNA. 293 cells were mock-transfected or transfected with in vitro-synthesized RNA transcripts encoding NSs or rLuc. Then the cells were mock-treated or treated with ActD. Total RNAs were harvested at 8 h post-transfection and the expression levels of PKR mRNA were examined by quantitative real-time reverse transcription polymerase chain reaction (RT-PCR) analysis (Figure 7C). The relative expression levels of PKR mRNA were significantly increased in cells that were transfected with rLuc RNA transcripts or NSs RNA transcripts both in the absence and presence of ActD, which demonstrated to us that NSs expression did not promote the degradation of PKR mRNA. Efficient ActD-mediated suppression of IFN-β mRNA accumulation in rMP-12-rLuc-infected 293 cells (Figure 6C) led us to suggest that unexpected increases in the abundance of PKR mRNA in the RNA-transfected 293 cells in the presence of ActD were IFN-β-independent. We suspect that the RNA transcripts that were taken up by the cells induced robust PKR mRNA synthesis prior to ActD- or NSs-mediated general transcription suppression.

Since NSs expression did not decrease the abundance of PKR mRNA (Figure 7C), we next tested whether putative NSs-mediated translational inhibition leads to a reduction in the abundance of PKR. To this end, 293 cells were mock-infected, infected with MP-12, or transfected with in vitro-synthesized RNA transcripts encoding NSs. Then cells were incubated with 100 µg/ml of puromycin to completely shut off cellular translation or puromycin untreated, and cell extracts were harvested at 16 h.p.i. or post-transfection. As expected, puromycin treatment completely abolished the synthesis of N and NSs proteins in MP-12-infected cells (Figure 7D, lane 4) and that of NSs in cells transfected with the RNA transcripts encoding NSs (Figure 7D, lane 6). We found that treatment of 293 cells with puromycin for 16 h decreased the abundance of PKR only slightly (Figure 7D, lane 2). Accordingly, it is highly unlikely that putative NSs-induced translational inhibition was the main reason for the reduction in PKR abundance.

We next performed pulse-chase experiments to know whether NSs expression promoted PKR degradation. Because immunoprecipitation experiments using various anti-PKR antibodies failed to convincingly demonstrate a radiolabelled endogenous PKR signal from extracts of 293 cells (data not shown), we examined the effect of NSs expression on the stability of an expressed mutant PKR, PKRK296R, lacking kinase activity [40] and carrying a N-terminal myc tag; expression of wild-type PKR was not used due to its strong host translation suppression effects (data not shown). 293 cells were mock-transfected or transfected with pcDNA3.1-Myc-PKRK296R, a plasmid encoding PKRK296R under cytomegalovirus promoter and radiolabelled with [^35^S] methionine/cycteine between 14 and 16 h post–DNA transfection. After pulse-radiolabelling cell extracts were prepared from some samples. In other samples, cells were transfected with in vitro-synthesized RNA transcripts encoding rLuc or NSs, and cell extracts were harvested at 8 h post–RNA transfection. Then the cell extracts were subjected to radioimmunoprecipitation analysis using anti-myc antibody, which immunoprecipitated the pulse-radiolabelled, myc-tagged PKR (Figure 7E, lane 2). After 8 h chase, the amount of the radiolabelled myc-tagged PKR was clearly reduced in the cells expressing NSs (Figure 7E, lane 4), but not those expressing rLuc (Figure 7E, lane 3). Instead, the amount of radiolabelled myc-tagged PKR was increased slightly in rLuc-expressing cells, presumably due to radiolabelling of continuously synthesized myc-tagged PKR by residual [^35^S] methionine/cysteine.

The complete shutoff of cellular translation by puromycin for 16 h did not result in the loss of endogenous PKR (Figure 7D), whereas as early as 8 h post-transfection of RNA transcripts encoding NSs, the abundance of PKR decreased substantially (Figure 7B) without reducing the abundance of PKR mRNA (Figure 7C). Furthermore, NSs expression reduced the abundance of myc-tagged PKR that had been radiolabelled prior to NSs expression (Figure 7E). These data strongly suggested that NSs induced the downregulation of PKR at a post-transcriptional level and pointed to a possibility that the NSs promoted PKR degradation.

We next tested whether RVFV NSs downregulated PKR by promoting PKR degradation through a ubiquitin-proteasome pathway. 293 cells were infected with rMP12-rLuc or MP-12, and were immediately treated with proteasome inhibitor MG132 or lactacystin. Cells were harvested at 8 h.p.i. and analyzed in Western blotting. As expected, rMP12-rLuc replication did not induce PKR downregulation (Figure 7F, lane 1), while MP-12 replication induced the reduction of PKR abundance (Figure 7F, lanes 4). It was evident that the treatment of MP-12-infected cells with those proteasome inhibitors suppressed NSs-induced PKR downregulation (Figure 7F, lanes 5 and 6), suggesting that NSs promoted PKR downregulation by the degradation through the proteasome pathway.

Phorbol 12-myristate 13-acetate (PMA), a potent activator of protein kinase C (PKC), induces PKR degradation [41]. Because the general PKC inhibitor, GÖ6983, suppresses PMA-mediated PKR degradation [41], we examined whether the NSs promoted PKR downregulation through PKC by treating MP-12-infected cells with GÖ6983. Treatment of GÖ6983 did not inhibit the NSs-mediated PKR downregulation (Figure 7F, lane 8), suggesting that PKC was not involved in NSs-induced PKR downregulation.

To know how quickly PKR downregulation occurred in MP-12-infected cells, whole-cell extracts were prepared at 2, 4, 6 and 8 h.p.i. from MP-12-infected 293 cells and the amounts of NSs and PKR were determined (Figure 7G). In the absence of proteasome inhibitor, a substantial reduction of PKR abundance occurred as early as 4 h.p.i., where the abundance of NSs (Figure 7G, lane 2) was not as great as that at 8 h.p.i. (Figure 7G, lane 4). In the presence of MG132, the abundance of PKR decreased only slightly, and NSs accumulation was also somewhat less efficient. A similar trend for less efficient NSs accumulation in the presence of proteasome inhibitors was also shown in the data for Figure 7F. Our observation of less efficient NSs accumulation in MG132-treated cells was consistent with the report that MG132 induces eIF2α phosphorylation through GCN2 activation and translational suppression [42]. The abundance of NSs in MG132-treated cells at 8 h.p.i. and that in untreated cells at 4 h.p.i. was similar, and yet PKR abundance in the former cells was clearly higher than that in the latter cells. Taken together, these data strongly suggested that RVFV NSs promoted PKR degradation through the proteasome-dependent pathway.

## Discussion

### Biological significance of NSs-mediated PKR downregulation

The present study explored a novel function of the RVFV NSs protein by testing the replication of RVFV lacking the NSs gene initially in type I IFN-incompetent VeroE6 cells [43],[44] in the presence of ActD or α-amanitin, which served as a surrogate of the host mRNA synthesis suppression function of the NSs. The NSs protein was essential for efficient virus replication in the presence of ActD or α-amanitin. We found that the replication of RVFV lacking the NSs gene in the presence of a transcription inhibitor induced an accumulation of phosphorylated eIF2α, The accumulation of phosphorylated eIF2α in the presence of transcriptional inhibitors, did not occur in VeroE6 cells that were infected with RVFV expressing a dominant-negative form of PKR (PKRΔE7) and in MEF cells lacking functional PKR (Figure 5). These findings suggested to us that PKR played a major role in increasing phosphorylated eIF2α; however, it is currently unclear whether other kinases, such as PERK or GCN2, have a possible role in eIF2α phosphorylation in rMP-12-rLuc-infected cells in the presence of transcriptional inhibitors. The endogenous PKR-mediated eIF2α phosphorylation suppressed translation of the RVFV lacking the NSs gene, resulting in poor virus replication. Our data further suggested that the NSs promoted PKR degradation most probably through a proteasome-dependent pathway and prevented eIF2α phosphorylation, leading to efficient viral translation.

The past studies [25],[26],[45] and the data shown in this study illustrate that two distinct biological activities of the RVFV NSs protein worked together to secure efficient RVFV replication. Namely, nuclear NSs protein inhibits transcription of host mRNAs, including the IFN mRNAs; this activity is critical for efficient RVFV replication in IFN-competent systems [25],[45]. However, a combination of RVFV replication and NSs-mediated host mRNA transcriptional suppression possibly induces PKR activation and subsequent eIF2α phosphorylation as a combination of RVFV replication and ActD or α-amanitin treatment induced it. The NSs protein, in turn, promotes PKR downregulation as early as 4 h.p.i., and prevents eIF2α phosphorylation to secure the translation of viral mRNAs and efficient virus replication. We think that both of these two NSs functions are tightly related and protect efficient viral replication by suppressing host antiviral responses. In RVFV-infected cells, the NSs establishes general transcriptional suppression at a later stage of infection (after 8 h.p.i.) [25], while NSs also suppresses specific IFN-β mRNA transcription at early stages of infection (about 3 h.p.i.) by maintaining repressor complex including SAP30 on IFN-β promoter [26],[45]. PKR downregulation early in infection is probably important for maintaining efficient viral translation in combination with the suppression of host IFN responses.

We suspect that the NSs-mediated PKR downregulation activity is important for RVFV replication and survival in infected mammalian hosts. RVFV-infection in rhesus monkeys showed that type I IFN is detectable around 1 day post-infection in both clinically ill surviving monkeys and lethally infected monkeys, and one dead monkey even kept high titer of IFN (120 to 480 U/ml) from 1 day post-infection [46]. In fact, the best correlation with outcome was early detection of IFN and not necessarily the height of the response. NSs suppresses IFN-β mRNA transcription early in the course of infection in cultured cells [26],[45]. Our present data suggest that the early downregulation of PKR in RVFV-infected cells might contribute to the inhibition of type I IFN induction, because PKR is known to serve as a pathogen-recognition receptor [47]. Furthermore, some of the host pathogen-recognition receptors, e.g., toll-like receptors 3 and 7, in uninfected cells located near the RVFV-infected cells, recognize virus-specific signals, e.g., viral RNAs in the virus particles or viral dsRNAs in the cell debris from infected cells, leading to type I IFN production. Because the transcriptional promoter of the PKR gene contains an IFN-stimulated response element, and IFN stimulation induces PKR mRNA transcription [38], PKR abundance is likely increased in many uninfected cells of infected animals; RVFV needs to replicate in such cells to survive in infected hosts. The reduction in PKR abundance occurred as early as 4 h.p.i. (Figure 7G) and the NSs protein is produced very early in RVFV infection [48]. Immediate NSs synthesis and subsequent NSs-induced PKR downregulation in the cells, some of which have increased PKR abundance, could rapidly disarm the PKR-mediated, antiviral functions and contribute to RVFV replication and survival in animal hosts.

### Possible mechanisms of the accumulation of phosphorylated eIF2α in ActD-treated, rMP12-rLuc–infected cells

ActD treatment of uninfected cells resulted in a significant reduction of the polysome fraction, but it did not abolish the translational activities (Figure S1). rMP12-rLuc replication in the cells treated with ActD or α-amanitin promoted the accumulation of the phosphorylated eIF2α, resulting in translational suppression of viral proteins (Figure 4). The increased amounts of phosphorylated eIF2α clearly correlated with the concentration of ActD or α-amanitin in rMP12-rLuc-infected cells (Figure S2). In contrast, rMP12-rLuc replication in the absence of transcriptional suppressor induced only low levels of eIF2α phosphorylation (Figure 4). Yet, the phosphorylation of PKR occurred in cells supporting the replication of RVFV lacking NSs both in the absence and presence of ActD (Figure 6D). Thus, a significant accumulation of phosphorylated eIF2α occurred only in cells in which replication of RVFV lacking NSs was combined with the treatment with transcriptional inhibitors.

Several different mechanisms are conceivable for the accumulation of phosphorylated eIF2α in rMP12-rLuc-infected in cells treated with ActD or α-amanitin (Figure 4). One possible mechanism relates to the eIF2α dephosphorylation step. Phosphorylation of eIF2α at Serine 51 induces a rapid synthesis of activating transcription factor (ATF)-4 mRNA, which can be translated in the presence of phosphorylated eIF2α [49]. Expressed ATF4 then induces GADD34 mRNA transcription [50] and GADD34 protein interacts with type 1 protein serine/threonine phosphatase, PP1, and this complex dephosphorylates eIF2α to resume the cellular translation [51]. rMP12-rLuc replication in transcriptionally active cells probably induced PKR activation and eIF2α phosphorylation, the latter of which then induced GADD34 upregulation and subsequent eIF2α dephosphorylation allowing efficient viral translation. rMP12-rLuc replication in cells treated with ActD induced PKR activation, the extent of which was similar to that of infected, ActD-untreated cells (Figure 6D), whereas the transcription inhibitors would prevent GADD34 upregulation and subsequent eIF2α dephosphorylation, causing an accumulation of phosphorylated eIF2α, which inhibited viral translation. Other possible mechanisms relate to the failure to suppress the PKR function. Cells infected with adenovirus, human immunodeficiency virus-1, or herpes simplex virus undergo a dramatic increase in the abundance of Alu RNA, which carries a repetitive element of ∼300-nt in length that is transcribed by RNA polymerase III [52],[53],[54],[55]. Alu RNA forms a stable complex with PKR, and antagonizes the PKR activation [56]. If rMP12-rLuc replication induces Alu RNA accumulation to prevent PKR activation, then the drug-induced transcriptional suppression would inhibit Alu RNA accumulation, preventing Alu RNA-mediated PKR inactivation. Another possibility is that rMP12-rLuc replication transcriptionally induces host mRNAs, and some of their gene products have PKR inhibition activities. An example of this possibility is that influenza virus replication induces P58^IPK^, an inhibitor of PKR [57]. ActD or α-amanitin treatment suppresses the expression of the putative PKR inhibitor, resulting in an accumulation of phosphorylated eIF2α. Alternatively, transcriptional suppression may result in reduced amounts of ribosomal proteins, such as L18, which binds to PKR and inhibits PKR activation [58],[59]. These possibilities are not mutually exclusive, and the combination of these possibilities may contribute to the accumulation of phosphorylated eIF2α in the cells supporting replication of RVFV lacking the NSs in the presence of ActD or α-amanitin.

### Possible mechanisms of the NSs-mediated PKR downregulation

Although many viruses suppress PKR function using various strategies [32], poliovirus is known to promote PKR degradation in infected cells [60],[61]; poliovirus RNA and viral protein components are required for this activity [61], and both eIF2α and PKR are highly activated in poliovirus-infected cells before PKR downregulation occurs [60]. In contrast to poliovirus, only RVFV NSs protein was probably required for PKR downregulation since the downregulation of PKR occurred as early as 4 h.p.i. in RVFV-infected cells (Figure 7G), whereas phosphorylated eIF2α accumulation occurred around 8 h.p.i. in rMP-12-rLuc-infected cells in the presence of ActD (Figure 4). Furthermore, NSs downregulated PKRK296R, a non-phosphorylatable mutant PKR (Figure 7E). These data suggest that the phosphorylation of PKR was not essential for the NSs-mediated PKR downregulation.

A substantial reduction in the abundance of PKR occurred in both the cytoplasmic and nuclear fractions of MP-12-infected cells, but not in the mock-infected cells and in those infected with MP12 lacking NSs (Figure 7). Expression of NSs alone resulted in a reduction in the amount of PKR (Figure 7B). These data established that NSs protein induced PKR downregulation. The NSs-induced PKR downregulation occurred as early as 4 h.p.i. of MP-12 (Figure 7G). We also demonstrated the presence of PKR mRNA in NSs-expressing cells (Figure 7C) and that ActD treatment had little effect on the NSs-induced PKR downregulation (Figure 7B). We failed to detect PKR at 16 h.p.i. of MP-12-infected 293 cells, and yet we easily detected the PKR after a 16 h-long incubation of 293 cells with puromycin (Figure 7D). Accordingly, the putative NSs-induced translational inhibition was not a main reason for the reduction in the abundance of PKR. Furthermore, pulse-chase experiments showed that NSs expression reduced the abundance of radiolabelled myc-tagged PKR (Figure 7E). All of these data strongly suggested that the NSs induced PKR downregulation at post-transcriptional levels.

It was reported that treatment of a macrophage cell line with IFN-γ induced PKR degradation [62]. Because NSs induced PKR degradation in ActD-treated cells and ActD treatment completely inhibited the IFN-γ mRNA accumulation (Figure 6C), it is highly unlikely that IFN-γ was involved in the NSs-induced PKR downregulation. PKC activation also potentially induces PKR downregulation [41]. Treatment of MP-12-infected cells with a PKC inhibitor, GÖ6983, did not inhibit NSs-mediated PKR downregulation (Figure 7D), which implied that PKC was not involved in it. Experiments using a proteasome inhibitor suggested that the NSs promoted PKR degradation through a proteasome pathway (Figure 7F). MG132 can induce GCN activation and translational suppression [42]. In fact, treatment of MP-12-infected cells with MG132 and lactacystin moderately reduced the accumulation of NSs (Figure 7F and 7G). Accordingly, a possibility still exists that the MG132-induced moderate translational inhibition affected the NSs-induced PKR degradation in MG132-treated cells. For example, if the NSs-induced PKR downregulation requires an unstable host protein, then the MG132-induced moderate translational suppression would prevent accumulation of this putative host protein, resulting in inhibition of the NSs-induced PKR downregulation. Obviously further studies are required to elucidate the mechanism of PKR downregulation mediated by RVFV NSs.

## Materials and Methods

### Cells and viruses

Vero E6 cells, wild-type mouse embryonic fibroblast (MEF) cells and *Pkr^0/0^* MEF cells [37] were maintained in Dulbecco's modified minimum essential medium (DMEM) (Invitrogen) containing 10% fetal bovine serum. BHK/T7-9 cells [63], which stably express T7 RNA polymerase, were grown in MEM-alpha (Invitrogen) containing 10% fetal bovine serum (FBS). Penicillin (100 U/ml) and streptomycin (100 µg/ml) (Invitrogen) were added to the media. BHK/T7-9 cells were selected in medium containing 600 µg/ml hygromycin (Cellgro). An RVFV vaccine candidate MP-12 [13] and recombinant MP-12 [19] were used for the experiments, and infectivity was assessed by a plaque assay in Vero E6 cells.

### Drug treatment

Cells were treated with transcriptional inhibitors, ActD (Sigma) (5 µg/ml) or α-amanitin (Sigma) (50 µg/ml) immediately after infection or transfection. To induce the inhibition of proteasome function, cells were immediately treated with MG132 (Sigma) at 10 µM or lactacystin (Sigma) at 50 µM after infection or transfection. To suppress PKC activity, cells were treated with a general PKC inhibitor, GÖ6983 (Calbiochem) at 100 nM immediately after infection with rMP12-rLuc or MP-12. Cells were treated with puromycin (Cellgro) at 100 µg/ml immediately after infection or transfection to inhibit cellular translation.

### Plasmid constructions

Standard molecular biological techniques, including a PCR-based mutagenesis method, were used for plasmid constructions. PCR fragments encoding PKRΔE7 ORF with an N-terminal Flag-tag sequence were cloned between the *Hpa* I and *Spe* I sites of pProT7-S(+) plasmid [19], designated as pProT7-S(+)-PKRΔE7. PCR fragments encoding the rLuc ORF or NSs with a C-terminal Flag-tag sequence were cloned between the *Hpa* I and *Spe* I sites of the pProT7-S(+) plasmid, designated as pProT7-S(+)-rLuc-Flag or pProT7-S(+)-NSs-Flag, respectively. All of the constructs were confirmed to have the expected sequences. The PCR product of the entire human PKR ORF carrying a point mutation at K296R and the N-terminal myc tag was cloned between *Kpn*I and *Xho*I of pcDNA3.1myc-His (Invitrogen), resulted in pcDNA3.1-Myc-PKR296R.

### Virus rescue

A recombinant MP-12 carrying the PKRΔE7 ORF, rLuc-Flag or the NSs-Flag in the place of the NSs ORF was recovered as described previously [19]. Briefly, subconfluent monolayers of BHK/T7-9 cells were co-transfected with an S-genome RNA expression plasmid, such as pProT7-S(+)-PKRΔE7, pProT7-S(+)-rLuc-Flag or pProT7-S(+)-NSs-Flag, and a mixture of pPro-T7-M(+), pPro-T7-L(+), pT7-IRES-vN, pCAGGS-vG, and pT7-IRES-vL using TransIT-LT1 (Mirus Bio Corporation). The culture medium was replaced with fresh medium 24 h later. At 5 days post-transfection, the culture supernatants were collected, clarified and then inoculated into VeroE6 cells. The supernatant of infected VeroE6 cells at 3 days post-infection was used for the experiment.

### RNA transfection

RVFV MP-12 or ZH501 NSs ORF, or CAT ORF were cloned downstream of the T7 promoter between the *Kpn* I and *Xho* I sites of the pcDNA3.1-myc-HisA (Invitrogen) plasmid. For rLuc RNA transcripts, the rLuc ORF in pRL-SV40 plasmid (Promega Corporation) was inserted downstream of the T7 promoter. Capped and polyadenylated RNA transcripts were synthesized in vitro by using mMESSAGE mMACHINE T7 Ultra (Ambion) according to the manufacturer's instructions [64]. One microgram of in vitro-synthesized RNA transcripts was transfected into 293 cells in a 12-well plate with a TransIT-mRNA Transfection kit (Mirus Bio Corporation) according to the manufacturer's instructions.

### Plaque assay

VeroE6 cells in 6-well plate were infected with a series of diluted virus samples in 400 µl. After 1 h adsorption at 37°C, we removed the inocula and added 2 ml of MEM containing 0.6% noble agar (Difco Laboratories), 5% FBS and 5% tryptose phosphate broth. The cells were incubated at 37°C for 3 days. Then, 2 ml of MEM containing 0.6% agar, 100 µg of neutral red (N2889, Sigma), 5% FBS and 5% tryptose phosphate broth were added into the wells, and incubated for 16 h. The virus titers were determined in triplicate.

### Western blot analysis

Cells were lysed in sample buffer and boiled for 10 min. Equal amounts of samples were subjected to sodium dodecyl sulfate-polyacrylamide gel electrophoresis (SDS-PAGE). Proteins were electroblotted onto polyvinylidene difluoride membranes (immobilon P; Millipore). Western blot was performed as described previously [27]. The following primary antibodies were used: anti-RVFV [27]; anti-NSs [48]; anti-eIF2α (#9722, Cell Signaling Tech.); anti–phospho-eIF2α (S51) (#9721, Cell Signaling Tech.); anti-Flag M2 (F3165, Sigma); anti-PKR (#3072, Cell Signaling Tech.); anti-Histone H1 (sc-8030, Santa Cruz Biotech.); anti–c-Myc (sc-40, Santa Cruz Biotech.), and anti–β-actin (sc-1616, Santa Cruz Biotech.).

### IP-kinase assay

The IP-kinase assay was performed as described previously [65]. Briefly, 293 cells were mock-infected or infected with recombinant MP-12 at an moi of 3. Cells were dissolved in lysis buffer containing 10 mM Tris-HCl pH 7.6, 50 mM KCl, 2 mM Magnesium acetate, 10 mM 2-mercaptoethanol, 1% Triton X-100, 1 mM EDTA, phosphatase inhibitor cocktail (Sigma) and proteasome inhibitor cocktail (Roche). After centrifugation at 10,000×*g* for 5 min, a cytoplasmic fraction was collected. Cytoplasmic lysates were subjected to immunoprecipitation with anti-PKR antibody (Santa Cruz Biotech, K-17). Protein A beads that bound to the immunoprecipitated PKR were washed twice with a buffer-A containing 20 mM Tris-HCl, pH 7.6; 50 mM KCl; 400 mM NaCl; 5 mM 2-mercaptoethanol; 1% Triton X-100; 1 mM EDTA; phosphatase inhibitor cocktail (Sigma); proteasome inhibitor cocktail (Roche); and 20% glycerol. The beads were further washed twice with buffer-B containing 20 mM Tris-HCl, pH 7.6; 100 mM KCl; 5 mM 2-mercaptoethanol; 1% Triton X-100; 0.1 mM EDTA; proteasome inhibitor cocktail (Roche); and 20% glycerol. Then, washed beads were resuspended in 2× kinase buffer containing 30 mM Hepes-KOH, pH 7.4; 2 mM dithiothreitol; 2 mM MgCl_2_; proteasome inhibitor cocktail (Roche); and 10 µCi of [γ-^32^P] ATP (MP Biomedicals). The suspension (20 µl) was incubated at 30°C for 20 min, and then 2× SDS sample buffer was added to terminate the reaction. The samples were separated on 10% SDS-PAGE gel and visualized on an autoradiograph. A portion of the samples were also used for Western blot analysis by employing anti-PKR monoclonal antibody (BD biosciences) to show the abundance of immunoprecipitated PKR.

### Radiolabelling of proteins

For the radiolabelling of host and viral proteins in infected cells, VeroE6 cells were incubated at 14.5 h post-infection for 30 min at 37°C with medium made up with MEM lacking methionine, cystine, and L-glutamine (M2289, Sigma); 1% dialyzed FBS (Invitrogen); 20 mM L-glutamine; penicillin (100 U/ml) and streptomycin (100 µg/ml). Then, Trans[^35^S]label metabolic reagent (MP biomedicals) was directly added into the medium (100 µCi/ml). After 1 h labelling, cells were washed with PBS once and lysed in sample buffer. Equal amounts of samples were subjected to SDS-PAGE in 10% polyacrylamide gel. The gel was dried and exposed to X-ray film (KODAK BioMax XAR) overnight at −80°C. For the radiolabelling of PKR, 293 cells were mock-treated or transfected with pcDNA3.1-Myc-PKRK296R, and labelled with Trans[^35^S]label metabolic reagent between 14 and 16 h post–DNA transfection. In some samples, cell extracts were prepared with chase using a lysis buffer. In other samples, cells were transfected with in vitro-synthesized RNA transcripts encoding rLuc or MP-12 NSs. Then, cells were washed, and incubated for 8 h in the presence of 2 mM methionine/cysteine. At 8 h post–RNA transfection, cell extracts prepared using lysis buffer, were employed for immunoprecipitation with anti-Myc antibody (Santa Cruz: sc-40), as described in an IP-kinase assay. Immunoprecipitated samples were separated on a 7.5% poryacrylamide gel and visualized on an autoradiograph.

### dsRNA-binding assay

293 cells were mock-infected or infected with rMP12-rLuc-Flag, rMP12-NSs-Flag or rMP12-PKRΔE7 at an moi of 3. Alternatively, 293 cells were transfected with in vitro-synthesized RNA transcripts encoding MP-12 NSs. The cytoplasmic lysate was harvested at 16 h.p.i. or 16 h post-transfection, and incubated with poly C beads or poly I∶C beads on ice for 45 minutes. After washing beads with buffer-A for 4 times, the beads were mixed with 2× sample buffer, and bound proteins were analyzed with anti-Flag antibody (Sigma) on a Western blot.

### *Renilla* luciferase assay

The luciferase assay was performed on a *Renilla* Luciferase Assay System (E2810, Promega Corporation) according to the manufacturer's instructions.

### Northern blot analysis

Total RNA was harvested by Trizol reagent (Invitrogen), and Northern blot was performed as described previously [27],[64]. Briefly, total RNA was denatured and separated on 1.2% denaturing agarose-formaldehyde gels and transferred onto a nylon membrane (Nylon Membrane, positively charged, Roche). Northern blot analysis was performed by using strand-specific RNA probes for detecting IFN-β mRNA [64], GAPDH mRNA [64], rLuc mRNA [27] or RVFV antiviral-sense S-segment / N-mRNA [48].

### Real-time PCR

293 cells in 6-well plates were transfected with 2 µg of in vitro-synthesized RNA transcripts encoding rLuc or MP-12 NSs by TransIT mRNA transfection kit (Mirus). Cells were mock-treated or treated with ActD (5 µg/ml) immediately after RNA transfection. Total RNA were extracted by using an RNeasy Mini kit (Qiagen) at 16 h post-transfection. For each sample, we used 500 ng of RNA to synthesize 1^st^ strand cDNA by High-Capacity cDNA Reverse Transcription Kits (Applied Biosystems). Real-Time PCR was performed at the Real-Time PCR core facility, Sealy Center for Cancer Cell Biology, UTMB. We used an Applied Biosystems made-to-order 20× assay mix of primers and TaqMan MGB probes (FAM dye-labled) for our target gene PKR (Applied Biosystems: assay ID#: Hs01091592_m1) and pre-developed an 18S rRNA (FAM-dye labelled probe) TaqMan assay reagent (Applied Biosystems: 4352930E) for endogenous control. Separate tubes (singleplex) real-time PCR was performed with 40 ng cDNA for both target gene and endogenous control by using a Taqman Gene expression master mix (Applied Biosystems: 4370074). The cycling parameters for real-time PCR were: UNG activation at 50°C for 2 min, AmpliTaq activation at 95°C for 10 min, denaturation at 95°C for 15 sec, and annealing/extension at 60°C for 1 min (repeat 40 times) on ABI7000. Duplicate C_T_ values were analyzed by the comparative C_T_ (ΔΔ C_T_) method, as described by the manufacturer (Applied Biosystems). The amount of target (2^−ΔΔCT^) was obtained by normalized to endogenous reference (18S rRNA) and relative to a calibrator (one of the experimental samples).

## Supporting Information

Figure S1 Effects of ActD treatment on host translation activities. (A) 293 cells cultured in 10-cm dishes were treated with 5 µg/ml of ActD (ActD) or were left untreated (Mock). Cells were harvested at 16 h post-ActD-treatment in 900 µl of Lysis buffer (50 mM Tris-HCl, pH 7.5; 5 mM MgCl2; 100 mM KCl; 1% Triton X-100; 100 µg/ml cycloheximide; and 0.5 mg/ml heparin) on ice for 5 min. The cytoplasmic lysates were collected after the removal of nucleus by centrifugation at 10,000×g for 5 min. The lysates were loaded onto a 10–50% linear sucrose gradient containing 50 mM Tris-HCl, pH 7.5; 5 mM MgCl2; 100 mM KCl; 0.5 mM dithiothreitol; 100 µg/ml cycloheximide; and 0.5 mg/ml heparin, and centrifuged at 38,000 rpm for 3 h at 4°C using a Beckman SW41 rotor. The gradients were pumped by syringe pump (Brandel) and analyzed by a density gradient fractionator (Brandel) connected to an ISCO UA-6 (ISCO Inc.) at the absorbance of 254 nm according to the manufacturer's instructions. The data were representative of two independent experiments. (B) 293 cells were transfected with in vitro-synthesized rLuc RNA transcripts and mock-treated (no drug) or immediately treated with 5 µg/ml of ActD (ActD) or 50 µg/ml of α-amanitin (Amanitin). Luciferase activities were measured at 16 h post-transfection. The data shown in the graphs (mean+/−standard deviation) were obtained from three independent experiments with p values by using Student's t-test (*: p<0.05).
(4.02 MB TIF)

Figure S2 Effects of different concentrations of ActD or α-amanitin on eIF2α phosphorylation and N protein accumulation in rMP-12-infected cells. VeroE6 cells were infected with rMP12-rLuc at an moi of 3, and then treated with different concentrations of ActD or α-amanitin. Cell extracts and culture supernatants were harvested at 16 h.p.i. Note that ActD suppresses about 80% of RNA polymerase I activity at 0.04 µg/ml, and about 80% of RNA polymerase II and III at 4.0 µg/ml [28], while α-amanitin suppresses nearly 100% of RNA polymerase II and about 50% of RNA polymerase III at 50 µg/ml [64]. (A) Western blot analysis of N protein, phosphorylated eIF2α, total eIF2α and α-actin in each cell extract. (B) Top panels represent the relative abundance of phosphorylated eIF2α and total eIF2α. The relative abundance of phosphorylated eIF2α and total eIF2α in the untreated cells represents 100%. The middle panels and the bottom panels represent the abundance of N protein and the virus titers, respectively. The results were obtained from three independent experiments with p values by using Student's t-test (*: p<0.05).
(4.33 MB TIF)

Figure S3 Effects of pan-caspase inhibitor Z-VADfmk on PKR-mediated eIF2α phosphorylation in infected cells. VeroE6 cells were mock-infected (Mock) infected with rMP12-rLuc (rMP12-rLuc) and then treated with 5 µg/ml ActD (Act) or 50 µg/ml of α-amanitin (Ama) or mock-treated (M) in the presence or absence of 100 µM of Z-VADfmk. Cells and culture supernatants were harvested at 16 h.p.i. (A) Western blot analysis of eIF2α, phosphorylated eIF2α, cleaved caspase 3 using anti-Cleaved Caspase 3, Asp175, antibody (Cell Signaling Tech. #9661), and α-actin in the absence (Top) and presence (Bottom) of Z-VADfmk. The data are representative of three independent experiments. (B) The relative abundance of phosphorylated eIF2α and total eIF2α. The relative abundance of phosphorylated eIF2α and total eIF2α in the mock-infected, mock-treated cells represents 100%. The average and standard deviation from three independent experiments were shown (*: p<0.05 compared to mock-treated cells). (C) Virus titers of rMP12-rLuc from three independent experiments were shown (*: p<0.05 compared to mock-treated cells).
(2.29 MB TIF)
