# Supplementary figures and images for: Rift Valley Fever Virus NSs Protein Promotes Post-Transcriptional Downregulation of Protein Kinase PKR and Inhibits eIF2α Phosphorylation
Source: PLoS Pathog. 2009 Feb 6;5(2):e1000287. doi: 10.1371/journal.ppat.1000287 (PMC2629125; doi:10.1371/journal.ppat.1000287)

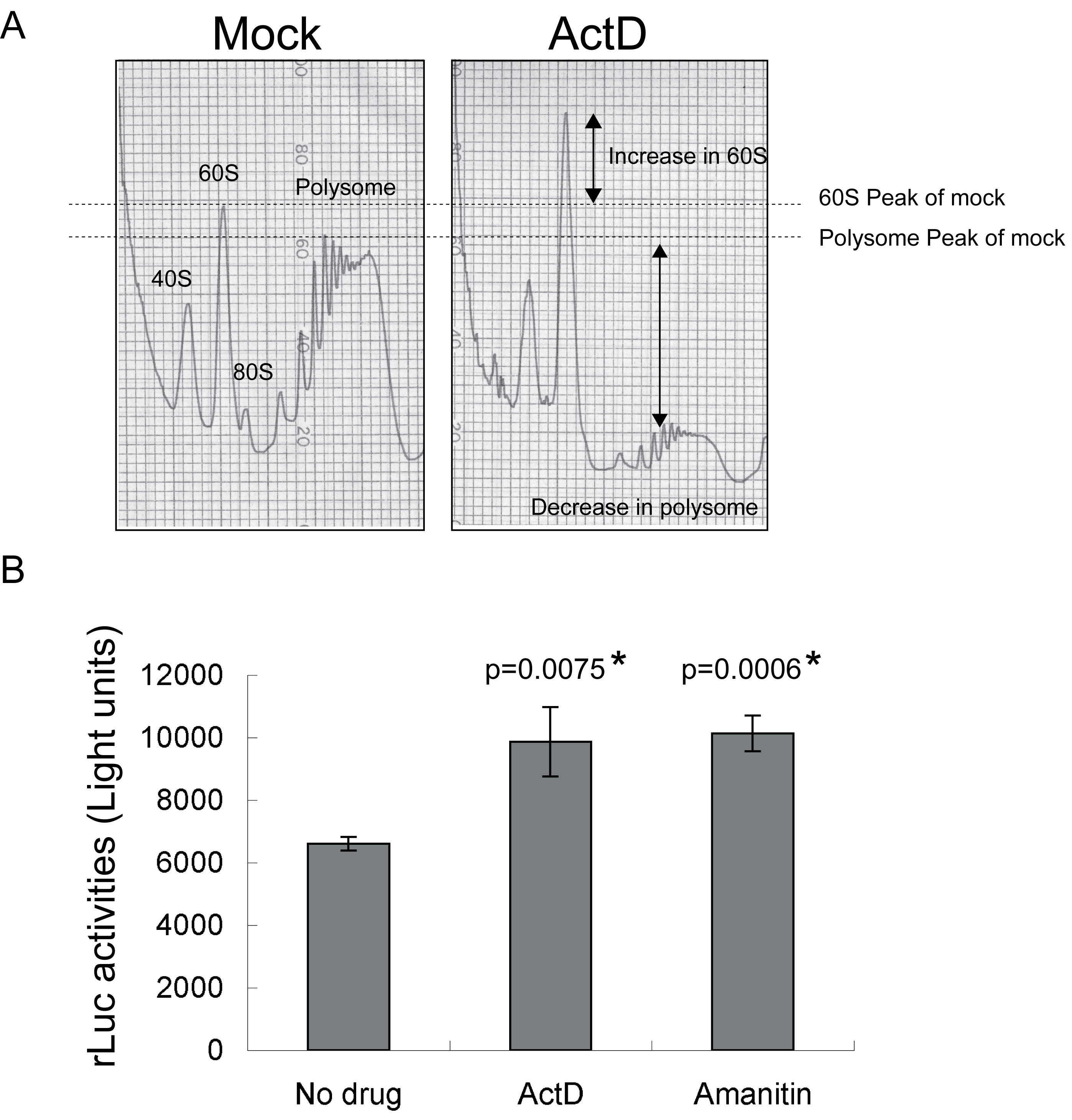

Supplement: Figure S1 — Effects of ActD treatment on host translation activities. (A) 293 cells cultured in 10-cm dishes were treated with 5 µg/ml of ActD (ActD) or were left untreated (Mock). Cells were harvested at 16 h post-ActD-treatment in 900 µl of Lysis buffer (50 mM Tris-HCl, pH 7.5; 5 mM MgCl2; 100 mM KCl; 1% Triton X-100; 100 µg/ml cycloheximide; and 0.5 mg/ml heparin) on ice for 5 min. The cytoplasmic lysates were collected after the removal of nucleus by centrifugation at 10,000×g for 5 min. The lysates were loaded onto a 10–50% linear sucrose gradient containing 50 mM Tris-HCl, pH 7.5; 5 mM MgCl2; 100 mM KCl; 0.5 mM dithiothreitol; 100 µg/ml cycloheximide; and 0.5 mg/ml heparin, and centrifuged at 38,000 rpm for 3 h at 4°C using a Beckman SW41 rotor. The gradients were pumped by syringe pump (Brandel) and analyzed by a density gradient fractionator (Brandel) connected to an ISCO UA-6 (ISCO Inc.) at the absorbance of 254 nm according to the manufacturer's instructions. The data were representative of two independent experiments. (B) 293 cells were transfected with in vitro-synthesized rLuc RNA transcripts and mock-treated (no drug) or immediately treated with 5 µg/ml of ActD (ActD) or 50 µg/ml of α-amanitin (Amanitin). Luciferase activities were measured at 16 h post-transfection. The data shown in the graphs (mean+/−standard deviation) were obtained from three independent experiments with p values by using Student's t-test (*: p<0.05). (4.02 MB TIF) [file ppat.1000287.s001.tif]

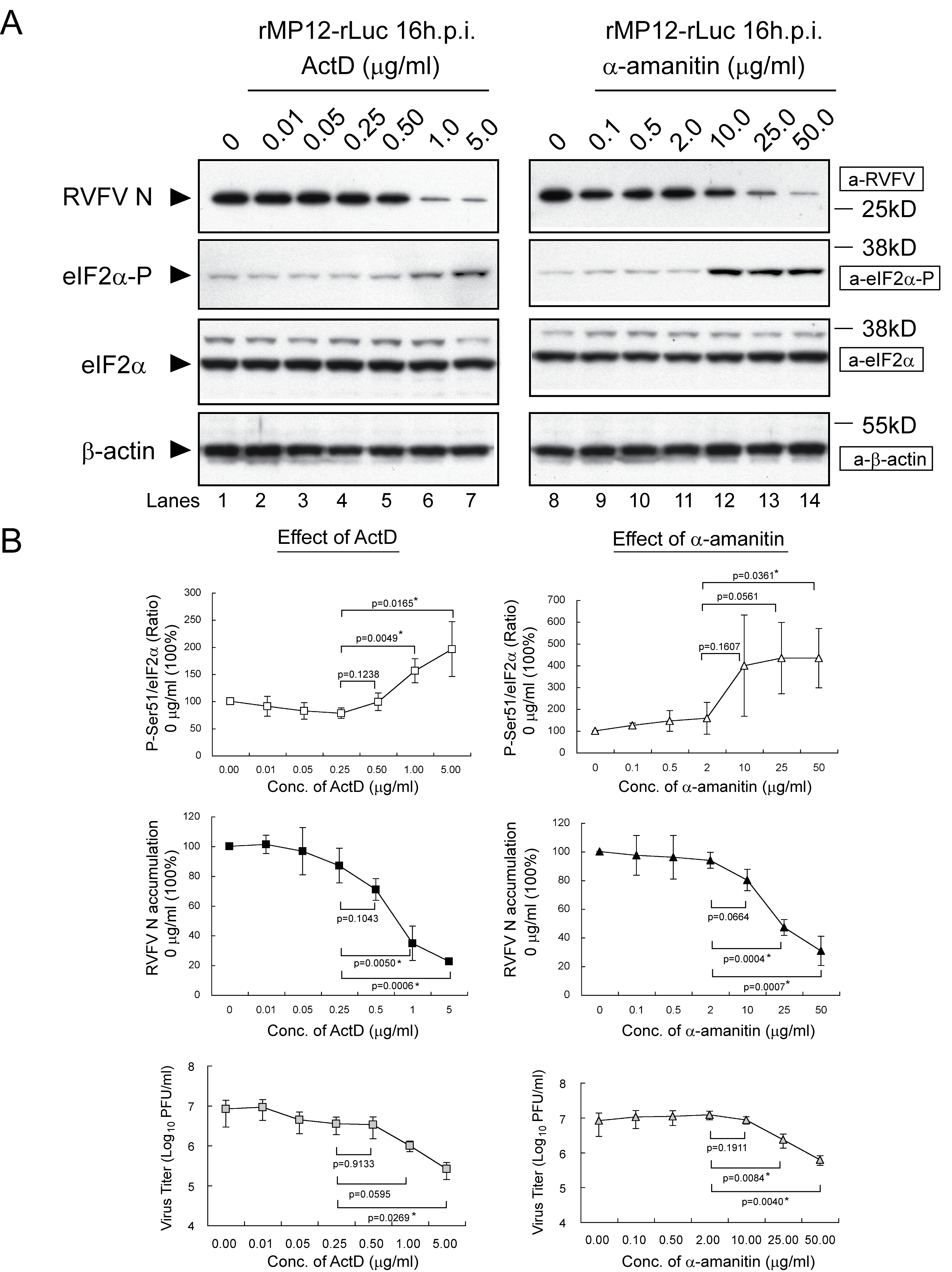

Supplement: Figure S2 — Effects of different concentrations of ActD or α-amanitin on eIF2α phosphorylation and N protein accumulation in rMP-12-infected cells. VeroE6 cells were infected with rMP12-rLuc at an moi of 3, and then treated with different concentrations of ActD or α-amanitin. Cell extracts and culture supernatants were harvested at 16 h.p.i. Note that ActD suppresses about 80% of RNA polymerase I activity at 0.04 µg/ml, and about 80% of RNA polymerase II and III at 4.0 µg/ml [28], while α-amanitin suppresses nearly 100% of RNA polymerase II and about 50% of RNA polymerase III at 50 µg/ml [64]. (A) Western blot analysis of N protein, phosphorylated eIF2α, total eIF2α and α-actin in each cell extract. (B) Top panels represent the relative abundance of phosphorylated eIF2α and total eIF2α. The relative abundance of phosphorylated eIF2α and total eIF2α in the untreated cells represents 100%. The middle panels and the bottom panels represent the abundance of N protein and the virus titers, respectively. The results were obtained from three independent experiments with p values by using Student's t-test (*: p<0.05). (4.33 MB TIF) [file ppat.1000287.s002.tif]

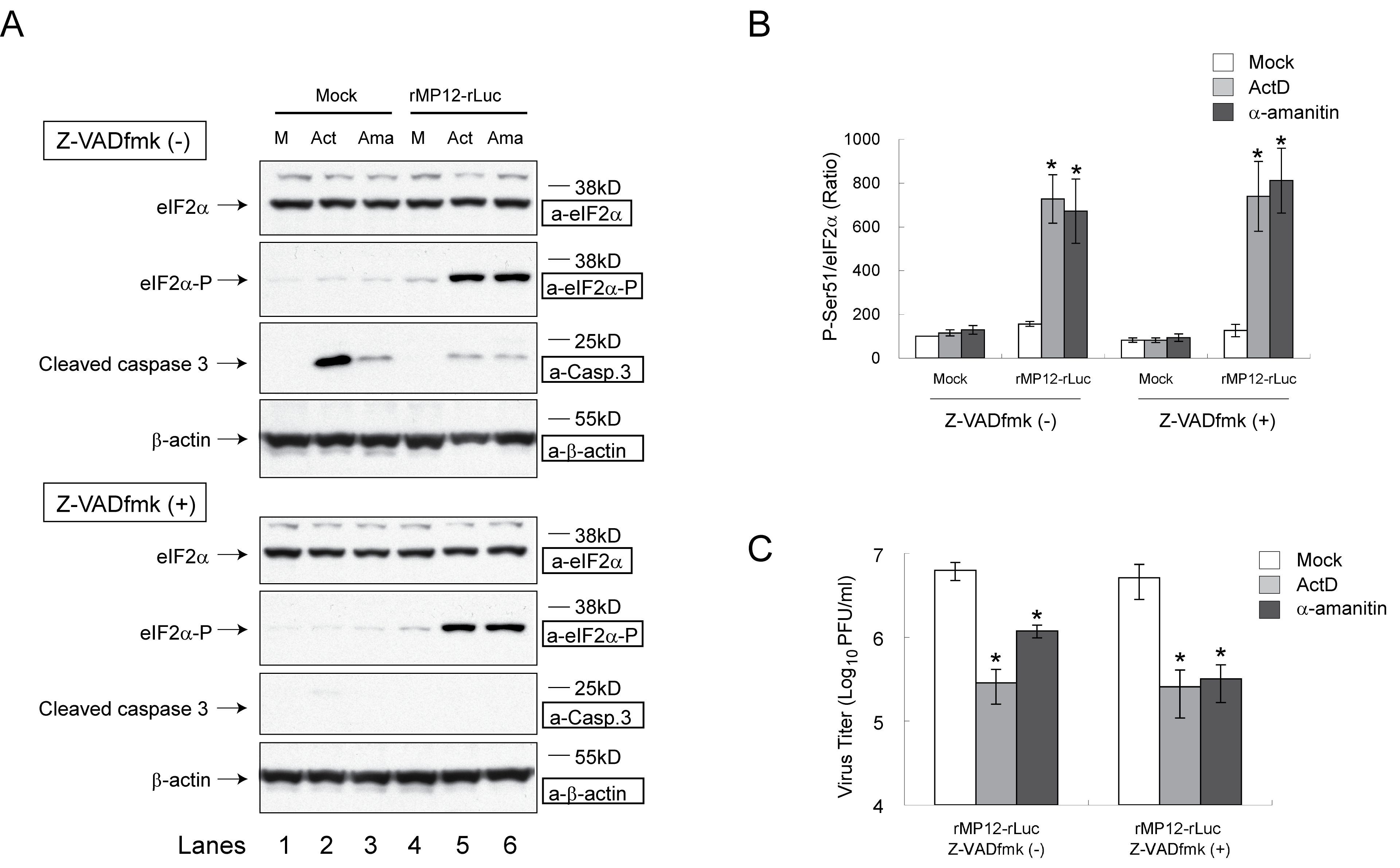

Supplement: Figure S3 — Effects of pan-caspase inhibitor Z-VADfmk on PKR-mediated eIF2α phosphorylation in infected cells. VeroE6 cells were mock-infected (Mock) infected with rMP12-rLuc (rMP12-rLuc) and then treated with 5 µg/ml ActD (Act) or 50 µg/ml of α-amanitin (Ama) or mock-treated (M) in the presence or absence of 100 µM of Z-VADfmk. Cells and culture supernatants were harvested at 16 h.p.i. (A) Western blot analysis of eIF2α, phosphorylated eIF2α, cleaved caspase 3 using anti-Cleaved Caspase 3, Asp175, antibody (Cell Signaling Tech. #9661), and α-actin in the absence (Top) and presence (Bottom) of Z-VADfmk. The data are representative of three independent experiments. (B) The relative abundance of phosphorylated eIF2α and total eIF2α. The relative abundance of phosphorylated eIF2α and total eIF2α in the mock-infected, mock-treated cells represents 100%. The average and standard deviation from three independent experiments were shown (*: p<0.05 compared to mock-treated cells). (C) Virus titers of rMP12-rLuc from three independent experiments were shown (*: p<0.05 compared to mock-treated cells). (2.29 MB TIF) [file ppat.1000287.s003.tif]
